# Mechanically Robust Lubricating Hydrogels Beyond the Natural Cartilage as Compliant Artificial Joint Coating

**DOI:** 10.1002/advs.202401000

**Published:** 2024-06-17

**Authors:** Weiyi Zhao, Yunlei Zhang, Xiaoduo Zhao, Wenbo Sheng, Shuanhong Ma, Feng Zhou

**Affiliations:** ^1^ State Key Laboratory of Solid Lubrication, Lanzhou Institute of Chemical Physics Chinese Academy of Sciences Lanzhou 730000 China; ^2^ Center of Materials Science and Optoelectronics Engineering University of Chinese Academy of Sciences Beijing 100049 China; ^3^ Shandong Laboratory of Advanced Materials and Green Manufacture at Yantai Yantai Zhongke Research Institute of Advanced Materials and Green Chemical Engineering Yantai 264006 China

**Keywords:** deformation recovery, high load‐bearing, low friction, mechanically robust hydrogels, wear‐resistance

## Abstract

Natural cartilage exhibits superior lubricity as well as an ultra‐long service lifetime, which is related to its surface hydration, load‐bearing, and deformation recovery feature. Until now, it is of great challenge to develop reliable cartilage lubricating materials or coatings with persistent robustness. Inspired by the unique biochemical structure and mechanics of natural cartilage, the study reports a novel cartilage‐hydrogel composed of top composite lubrication layer and bottom mechanical load‐bearing layer, by covalently manufacturing thick polyelectrolyte brush phase through sub‐surface of tough hydrogel matrix with multi‐level crystallization phase. Due to multiple network dissipation mechanisms of matrix, this hydrogel can achieve a high compression modulus of 11.8 MPa, a reversible creep recovery (creep strain: ≈2%), along with excellent anti‐swelling feature in physiological medium (v/v_0_ < 5%). Using low‐viscosity PBS as lubricant, this hydrogel demonstrates persistent lubricity (average COF: ≈0.027) under a high contact pressure of 2.06 MPa with encountering 100k reciprocating sliding cycles, negligible wear and a deformation recovery of collapse pit in testing area. The extraordinary lubrication performance of this hydrogel is comparable to but beyond the natural animal cartilage, and can be used as compliant coating for implantable articular material of UHMWPE to present, offering more robust lubricity than current commercial system.

## Graphical abstract

A novel layered cartilage‐inspired hydrogel is prepared by covalently manufacturing a thick composite lubrication layer through the sub‐surface of the mechanically robust hydrogel matrix. This hydrogel demonstrates good/durable lubricity (COF: 0.02–0.03, 100k cycles) in PBS medium under high contact pressure (*P* ≈2.5 MPa) due to the multi‐dissipation mechanism, comparable to natural bovine cartilage. This cartilage‐inspired hydrogel can be used as a robust lubrication coating for UHMWPE substrate.

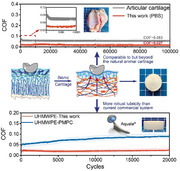

## 1 Introduction

Articular cartilage exhibits lasting and recoverable lubrication performance in the decades of human body service.^[^ ^1^ ^]^ Its internal matrix consists of multiple, orderly arranged layers of collagen,^[^ ^2^ ^]^ providing the cartilage with excellent tensile stiffness and compressive strength.^[^ ^3^ ^]^ Additionally, a large number of brushed‐biomacromolecules and lipid molecules arranged on its superficial zone endow cartilage with strong hydration effect.^[^ ^4^ ^]^ This unique combination of bulk structure and surface chemistry results in an extraordinary bio‐lubricated system with excellent load‐bearing, lubrication, anti‐wear, and rehydration properties.^[^ ^1^, ^5^ ^]^ Among them, the anti‐creep feature, fatigue‐resistance and deformation recovery after encountering dynamic loading/shearing are crucial for maintaining the durable lubricity of the cartilage layer.^[^ ^6^ ^]^ However, due to the poor physiological recovery ability resulting from eldering, disease, and other factors, cartilage systems always fail to lubricate and can inevitably be damaged. It is essential to develop artificial cartilage lubricating materials for solving this problem.

Hydrogels with strong surface hydration and low coefficient of friction (COF) have been investigated as potential replacement materials for damaged cartilage.^[^ ^1^, ^7^ ^]^ Even though recent researches for synthetic cartilage hydrogels based on double‐crosslinked network (DN) system have achieved significant progress,^[^ ^7c–f^ ^]^ the inherent anti‐swelling performance and biocompatibility in physiological media is still challenging. Among them, poly (hydroxyethyl methacrylate) (pHEMA) and polyvinyl alcohol (PVA) hydrogels are the most representative materials due to their excellent biocompatibility and medium stability.^[^ ^8^ ^]^ Furthermore, recent research indicated that incorporating trace lipids into the pHEMA hydrogel network can significantly improve its load‐bearing, lubrication, and wear resistance, resulting from a wear‐induced progressive release mechanism.^[^ ^8a^ ^]^ However, the lubrication lifetime may not be ideal due to the limitation of lubricant doping content. In fact, the inherent poor mechanical properties of pure pHEMA hydrogels such as low compression strength, irreversible creep recovery, and serve contact deformation under large‐loads condition, limit its lubrication performance in bioengineering applications.^[^ ^9^ ^]^ Compared to pHEMA hydrogels, PVA hydrogels show a hydrated surface and good elastic recovery after encountering contact deformation.^[^ ^7^, ^10^ ^]^ However, their low moduli commonly lead to poor load‐bearing capacity and obvious deformation contribution in the friction. Even though researchers have used diverse strategies to vastly improve their mechanical properties, such as annealing treatment,^[^ ^11^ ^]^ nanocomposite reinforcement,^[^ ^7c^ ^]^ mechanical training,^[^ ^12^ ^]^ and salting out,^[^ ^8^, ^13^ ^]^ the strength enhancement is always at the expense of surface hydration.^[^ ^10^, ^14^ ^]^ and lubrication performance, which is contrary to achieving a balance between strength and lubrication in natural cartilage.^[^ ^7^, ^15^ ^]^ Until now, developing a mechanically robust cartilage‐like hydrogel system suitable for biological medium with low COF, high load‐bearing capacity, and deformation recovery simultaneously is still challenging.

Here, we developed a novel cartilage‐inspired layered lubrication hydrogel (H_PVA/CS‐PSPMA_) by covalently manufacturing thick (several tens of micrometers) poly(3‐sulfopropyl methacrylate potassium) (PSPMA) polyelectrolyte brushes (PB) phase through the sub‐surface of a robust polyvinyl alcohol/chitosan (PVA/CS) hydrogel matrix (H_PVA/CS_) with multi‐level crystallization networks. The top lubrication layer shows excellent lubricity and wear resistance, whereas the nanocrystalline domain‐enhanced H_PVA/CS_ matrix exhibits good load‐bearing, anti‐swelling, and deformation recovery properties. The lubrication performance of H_PVA/CS‐PSPMA_ is even better than that of the natural cartilage under the same test condition and could be used as a robust and compliant coating for implantable material of UHMWPE (ultrahigh molecular weight polyethylene), which presents a great advantage for replacing the traditional thin polyelectrolyte brushes lubrication layer. Based on these results, this strategy provides a new design routine for cartilage‐like hydrogels where surface lubrication and bulk mechanical strength merge well.

## 2 Results and Discussion

### 2.1 Design Concept and Preparation of Cartilage Hydrogels (H_PVA/CS‐PSPMA_)

**Figure 1:**
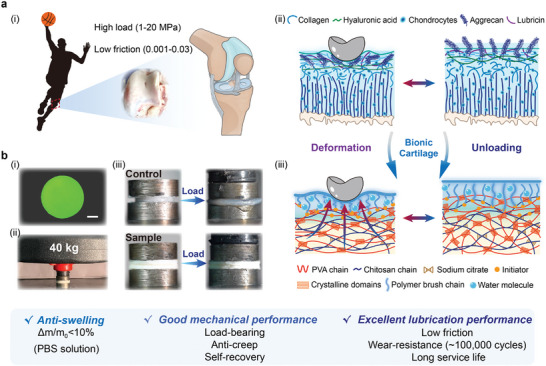
Design concept of bionic cartilage hydrogel H_PVA/CS‐PSPMA_. a) Schematic diagrams of the human articular cartilage system (i), reversible mechanical deformation and recovery behavior of natural cartilage layer composed of hydrated superficial layer and robust load‐bearing matrix under loading/unloading process (ii), and reversible mechanical deformation and recovery feature of bionic layered cartilage hydrogel (H_PVA/CS‐PSPMA_) composed of top composite lubrication layer and bottom mechanical support layer under loading/unloading process (iii). b) (i) Fluorescence photograph of H_PVA/CS‐PSPMA_. (Scale bar: 5 mm) (ii) H_PVA/CS‐PSPMA_ bears the weight of a 40 kg dumbbell. iii) Photographs of H_PVA/CS‐PSPMA_ and control hydrogel during compression process with 40 kg.

Natural articular cartilage exhibits low COF (0.001–0.03), durable lubricity (several decades), and dynamically mechanical recovery under high contact stresses (1–20 MPa),^[^ ^16^ ^]^ which are highly related to its typical layered structure, good mechanical property and efficient surface hydration (**Figure** **1**a(i)). Among them, the highly hydrated hydrophilic macromolecules anchored onto the cartilage surface are mainly responsible for friction‐reduction and wear resistance, while the reversibly mechanical recovery feature of the deformable cartilage layer under cyclically loading and shearing process,^[^ ^5a^ ^]^ is the prerequisite for resisting fatigue and maintaining its ultra‐long lifetime (Figure 1a(ii)). Inspired by these mechanisms, a layered cartilage hydrogel (H_PVA/CS‐PSPMA_) composed of a top soft lubrication layer and a bottom load‐bearing matrix, was prepared by covalently manufacturing hydrophilic PSPMA polyelectrolyte brushes phase through the sub‐surface of mechanically robust PVA/CS hydrogel (H_PVA/CS_) (Figure 1a(iii)). The detailed preparation process of H_PVA/CS‐PSPMA_ was shown in Figures S1 and S2 (Supporting Information). In typical case, H_PVA/CS_ matrix surface was covalently anchored with thick atom transfer radical polymerization (ATRP) initiator layer to obtain H_PVA/CS‐Br_,^[^ ^17^ ^]^ and then the composite lubrication layer was generated by employing an in‐situ permeation‐accelerated polymerization strategy for spatially grafting of hydrophilic PSPMA brushes chains through its sub‐surface.

Meanwhile, the H_PVA/CS_ matrix was constructed by employing nanocrystalline domain‐enhancement strategy with multiple physically crosslinking networks (Figure S3, Supporting Information), including the first hydrogen bonding network of PVA/CS or CS/CS, the second ion coordination network of sodium citrate (Na_3_Cit)/CS with additional salting out effect, and the third hydrogen bonding network between chains of PVA molecules by freeze‐thaw and annealing crystallization. The typical multiple physically crosslinking feature of the H_PVA/CS_ matrix endowed it with multi‐level crystallization networks and good mechanical robustness, as well as excellent load‐bearing and dynamically deformation recovery feature as natural cartilage layer. As a result, the negatively charged PSPMA chains in the top composite lubrication layer can mimic the brush‐like proteoglycan chains on the superficial layer of cartilage, acting as the efficient lubrication phase, while the bottom H_PVA/CS_ matrix possessed excellent load‐bearing and anti‐fatigue functionalities. Therefore, the H_PVA/CS‐PSPMA_, a novel cartilage hydrogel, would present reversibly mechanical deformation‐recovery feature under dynamic loading/unloading processes (Figure 1a(iii)), which enables to achieve lasting lubrication.

Fluorescence photograph of H_PVA/CS‐PSPMA_ hydrogels sample was shown in Figure 1b(i). The H_PVA/CS‐PSPMA_ sample with a diameter of 20 mm only showed a low strain of <8% against 40 kg (compressive stress of 1.25 MPa, in range of contact stress level of articular cartilage.^[^ ^7c^ ^]^) weight, compared to the traditional PVA/CS hydrogels (control sample) presenting large mechanical deformation (Figure 1b(ii,iii)). This implied that H_PVA/CS‐PSPMA_ hydrogels possessed considerable load‐bearing capacity as cartilage‐like matrix. Overall, based on novel bionic design concept, our current H_PVA/CS‐PSPMA_ exhibited excellent anti‐swelling feature in physiological medium, good mechanical properties (load‐bearing, anti‐creep, and self‐recovery), and excellent lubrication performance (low friction, wear‐resistance, and long service life).

### 2.2 Characterizations of Structure and Anti‐Swelling Properties of H_PVA/CS_ Matrix

**Figure 2:**
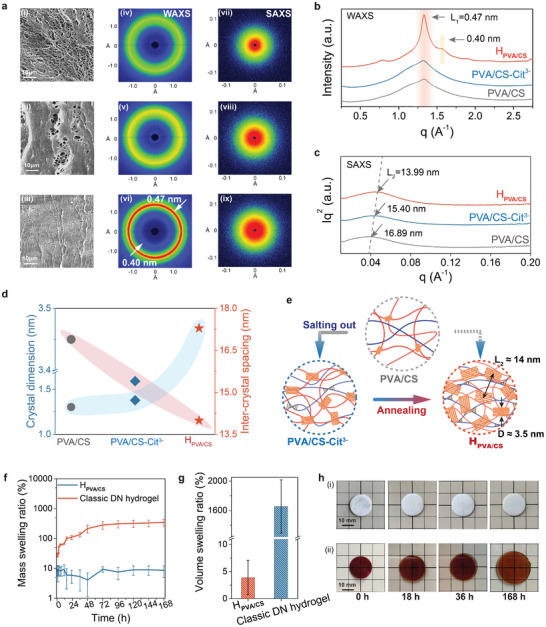
Characterizations of microstructure and anti‐swelling properties. a) SEM images, 2D WAXS, and SAXS debye scattering rings of the hydrogel matrix after different treatments: (i, iv, vii) PVA/CS, (ii, v, viii) PVA/CS‐Cit,^3^ ^−^ (iii, vi,ix) H_PVA/CS_. b) The integrated spectra of WAXS profiles were plotted as a function of the scattering vector, intensity∼q. c) The integrated spectra of SAXS profiles were plotted as a function of the scattering vector, intensity*q^2^∼q. d) The crystal dimension and inter‐crystal spacing data of different hydrogel matrixes. e) Schematic diagram of changes in microscopic crystal regions of different hydrogels. f) The mass swelling ratio‐immersing time curves in PBS buffer solution (pH 7.4) of H_PVA/CS_ sample and classic DN hydrogel (Fe‐P(AAm/AAc) hydrogel) system. g) The volume swelling ratios in PBS (pH 7.4) buffer solution of H_PVA/CS_ sample and classic DN hydrogel. h) The swelling photographs of H_PVA/CS‐PSPMA_(i) and Fe‐P(AAm/AAc) hydrogel (ii) after immersing into PBS for different time (Scale bar: 10 mm). Data in (F) and (G) are means ± SD, n = 3.

The mechanisms responsible for mechanical robustness of H_PVA/CS_ matrix were investigated. **Figure** **2**a showed the corresponding surface morphology and micro‐structure characterizations of the as‐prepared hydrogels samples in different stages. By jointly deploying multi‐physical interactions and subsequent annealing treatment, networks of hydrogel samples showed different chains stacking densities. Traditional PVA/CS hydrogels matrix was loose and porous, while the multi‐step treated H_PVA/CS_ was dense (Figure 2a(i–iii)). Dense entanglement of polymer chains and effective dissipation mechanisms due to multiple physical networks contributed to the overall high stiffness and toughness of the H_PVA/CS_ hydrogel.^[^ ^6^, ^12^ ^]^ The multiple physically crosslinking networks within H_PVA/CS_ matrix could be confirmed by Fourier Transform infrared spectroscopy (FT‐IR) (Figure S4, Supporting Information). Compared to the PVA/CS hydrogels and PVA/CS‐Cit3^−^ hydrogels (without annealing treatment), the obvious shift of stretching vibration peak of hydroxyl groups (‐OH) toward low wavenumber (cm^−1^) for H_PVA/CS_ indicated the successful network enhancement by hydrogen bonding interactions. The signal appeared at 1142 cm^−1^ belongs to the typical crystalline phase,^[^ ^10c^ ^]^ indicating that crystallinity could effectively be improved by employing salting out and annealing means. The crystallinities of different samples were characterized by X‐Ray Diffraction (XRD) and Differential Scanning Calorimeter (DSC) (Figures S5 and S6, Supporting Information). Compared to the untreated PVA/CS, the gradual increase of signal strength at $10\bar{1}$ peak (2θ = 20°) of PVA chains for PVA/CS‐Cit3^−^ hydrogels and H_PVA/CS_ was observed, indicating the existence of a crystalline micro‐domain from synergism of ions coordination, salting out and annealing treatments. Besides, further annealing treatment for strengthening the hydrogen bonding interaction between PVA chains resulted in the continuous increase of the network crystallinity as well as the shrinkage of porosity.

Detailed crystallization enhancement mechanisms within hydrogel networks were explored by using Wide Angle X‐Ray Scattering (WAXS) and Small Angle X‐Ray Scattering (SAXS). Through X‐ray scattering tests, the details of the internal micro‐structures within the hydrogel matrix can be detected. As shown in Figure 2a(iv–vi), compared to the PVA/CS sample, the H_PVA/CS_ sample showed significant diffraction rings, and the intensity data was further plotted and analyzed (Figure 2b). The 2D WAXS patterns showed a clear Debye scattering ring with maximum intensity at q = 1.33 Å^−1^, indicating the existence of higher crystallinity. Moreover, the other weaker scattering ring corresponded to q = 1.57 Å^−1^. The distinct peak at q = 1.33 Å^−1^ revealed the lattice spacing (L_1_) of 0.47 nm, which may be correlated to the ordered hydrogen bonding network structure between the hydroxyl groups (‐OH) of PVA molecules. An additional peak appeared at q = 1.57 Å^−1^ with a lattice spacing of 0.4 nm, which was associated with the Cα‐Cα spacing of the hydrophobic domains of the CH_2_‐CH_2_ backbone.^[^ ^18^ ^]^ Such a lattice (0.4 nm) might originate from the hydrogen bond‐stabilized spacing between two CH_2_‐CH_2_ backbones. At the same time, the SAXS results showed that the inter‐crystal spacing within the hydrogels networks became smaller after the multi‐step crystallization treatment (Figure 2a(vii–ix),c). After salting out and annealing treatment, the crystal dimension (D) increased from 1.27 to 3.47 nm, while inter‐crystal spacing (L_2_) decreased from 16.89 to 13.99 nm (Figure 2d). This increase in the size of the nanocrystalline domain and a decrease in the distance between them led to the crystallinity increasing (Figure 2e).

As a result, the multiple physically crosslinking and crystallinity networks of H_PVA/CS_ could effectively improve its anti‐swelling property. Especially, the anti‐swelling property of cartilage‐like hydrogels materials was crucial due to their fluid‐encountered application environment. In addition, as long‐term potential implant material and coating, it was essential to maintain structure stability of hydrogels in the physiological medium over time. Therefore, the media‐stability of H_PVA/CS_ was investigated in PBS buffer solution (pH 7.4), while the classical double crosslinking networks (DN) hydrogels system (Fe‐P(AAm/AAc)) was used as comparison.^[^ ^19^ ^]^ The swelling process was real‐time recorded by monitoring the mass and volume change ratio (swelling ratio) of samples within 168 h (7 days) (Figure 2f,g). The Fe‐P(AAm/AAc) hydrogels swelled rapidly within 48 h and then gradually achieving equilibrium after 72 h, and the volume swelling ratio was obviously more than 1600%. By contrast, the anti‐swelling behavior of H_PVA/CS_ was excellent (mass swelling ratio <10%, volume swelling ratio <5%), which could be attributed to the hydrogen bonding‐enabled network crystal effect.^[^ ^15c^ ^]^ The crystallinity enhancement of H_PVA/CS_ enabled the obvious increase of density for the sample interior network, while the decrease of porosity made the whole material more hydrophobic and stable (Figure 2h). These results indicated the successful preparation of the mechanically robust H_PVA/CS_, suggesting that it can be used as a load‐bearing matrix for cartilage‐like hydrogels.

### 2.3 Evaluation of the Mechanical Properties of H_PVA/CS_ Matrix

**Figure 3:**
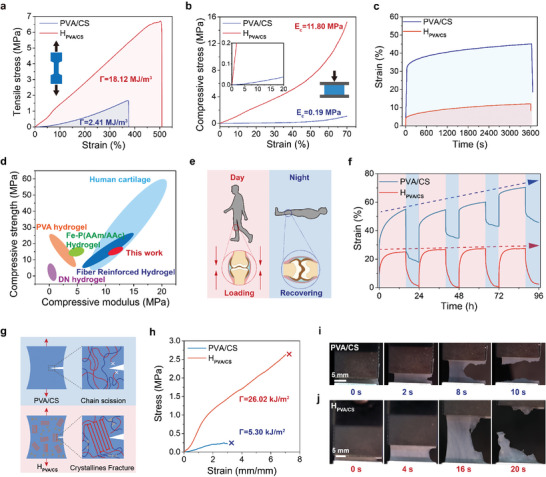
Characterizations of the mechanical properties. a) Tensile stress–strain curves of PVA/CS and H_PVA/CS_. b) Compression stress‐strain curves of PVA/CS and H_PVA/CS_ (the maximum strain was limited to 70%). c) Creeping curves of PVA/CS and H_PVA/CS_. d) Ashby diagram of compress strengths versus compress moduli of H_PVA/CS_ and some reported hydrogels systems (i.e., freeze‐thawed PVA hydrogel,^[^ ^10^, ^23^ ^]^ Human cartilage,^[^ ^6^, ^7^, ^24^ ^]^ Fe‐P(AAm/AAc) hydrogel,^[^ ^19^, ^25^ ^]^ DN hydrogel,^[^ ^26^ ^]^ Fiber Reinforced hydrogel.^[^ ^7c^ ^]^). e) Schematic diagram showing the cyclic process of daytime loading (pink area) and nighttime recovering (blue area) of the natural cartilage layer. f) Four cyclic strain‐time curves of PVA/CS and H_PVA/CS_ under dynamic creep loading (pink area, 16 h)/unloading (blue area, 8 h) process. g) Schematic diagram of the tensile tearing test of the sample and anti‐fracture mechanism. h) Tensile stress‐strain curves for the tearing test of PVA/CS and H_PVA/CS_. Snapshots of i) PVA/CS sample and j) H_PVA/CS_ in real‐time fracture process (Scale bar: 5 mm).

In order to validate our design concept for achieving mechanically robust H_PVA/CS_, a series of mechanical tests were performed on PVA/CS samples (the hydrogel without salting out and annealing treatment) and H_PVA/CS_ samples. All of the tests were performed after the samples encountering dynamic swelling equilibrium in PBS solution. First of all, the mechanical properties of the samples were systematically investigated by employing macro‐mechanical tests mean. The tensile stress–strain curves of the samples were shown in **Figure** **3**a. For PVA/CS, the elongation and tensile strength at break were 372% and 1.67 MPa, while those of H_PVA/CS_ were 503% and 6.7 MPa, separately. Correspondingly, the elastic modulus and the tensile toughness (Γ) were 0.07 MPa and 2.41 MJ m^−3^ for the PVA/CS sample, while those of H_PVA/CS_ were 1.32 MPa and 18.12 MJ m^−3^, separately (Figure S7, Supporting Information). The compressive property was crucial for lubrication materials in friction process, because it reflected the ability to resist mechanical deformation. The compressive modulus of the H_PVA/CS_ is 11.8 MPa that was nearly two orders of magnitude higher than that of PVA/CS sample (0.19 MPa) (Figure 3b). At the same compression strain of 70%, the compression strength of PVA/CS sample was only 1.09 MPa (toughness: 0.09 MJ m^−3^), while that of H_PVA/CS_ was as high as 15.25 MPa (toughness: 3.38 MJ m^−3^). These results indicate that H_PVA/CS_ possesses excellent load‐bearing capacity after employing salting out and annealing treatment means.

However, the short‐time compression test could not fully reflect the real loading‐condition during friction process. Hydrogel materials commonly tend to produce creep under continuous loading process, and then irreversible deformation and failure were occurred. The anti‐creeping ability of the hydrogel was a key factor to dominate its load‐bearing capacity and lubrication performance. Through a single creep test (5 N, 3600 s), it was found that the creeping strain of the PVA/CS sample was about twice that of the H_PVA/CS_ sample, confirming that our multi‐level crystallization strategy could well suppress the creep (Figure 3c). Both the compression strength and compression modulus of H_PVA/CS_ were superior to most reported hydrogels systems, and comparable to that of natural cartilage (Figure 3d). The excellent load‐bearing capacity of the H_PVA/CS_ was attributed to its high mechanical strength from nanocrystalline domains by dense polymer chains and effective dissipation mechanism from multiple physically crosslinking networks, for which could effectively improve the overall stiffness and toughness to resist deformation. Obviously, both the compression strength and modulus of H_PVA/CS_ were superior to most reported hydrogel systems and comparable to that of natural cartilage.^[^ ^6^, ^12^ ^]^

Furthermore, creep‐resistance and mechanical recovery features were also of paramount importance for achieving lasting high load‐bearing capacity. Correspondingly, the ability to maintain minor compression strain and recover to the original state after encountering deformation of samples was evaluated. In order to simulate the situation of human's joint in real life (Figure 3e), a 16 h creep versus 8 h recovery experiment was conducted to analogize the process of daytime loading and nighttime recovery of articular cartilage. The cyclic creep‐recovery curves of the PVA/CS sample and H_PVA/CS_ at 5 N normal load (equivalent contact stress was 0.2 MPa, in line with the range of articular cartilage.^[^ ^7c^ ^]^) at room temperature for 96 h, were shown in Figure 3f. In the creep process, the viscoelasticity of the samples caused the nonlinear variation of mechanical deformation with time. The creep deformation increased rapidly after being subjected to instantaneously loading at the beginning, then the creep rate gradually slowed down due to the slow deformation of polymer chains over time. By comparing the normal strain values from creep‐recovery curves of PVA/CS and H_PVA/CS_, it was found that the creep deformation of H_PVA/CS_ was smaller and remained unchanged after reaching equilibrium. After four creep‐recovery cycles, the net deformation strain value of H_PVA/CS_ was only 2%. As an intuitive contrast, the net deformation strain value of the PVA/CS sample was 45% after encountering four cycles. The mechanism responsible for this could be attributed to that the salting out and annealing treatment enabled the densification of the PVA/CS hydrogels networks, which largely restricted molecular chains movement and improved the ability of H_PVA/CS_ to resist creep deformation.

The nanocrystalline domains also act as strong crosslinking sites within H_PVA/CS_ networks to resist the irreversible deformation as well as enhance the deformation recovery. Such typical recovery feature was essential for achieving ultra‐long lubricity and load‐bearing during friction process. Fracture resistance was also one of the most important properties of the synthetic cartilage hydrogels. As shown in Figure 3g,h, H_PVA/CS_ exhibited high fracture stress of ≈2.8 MPa and a large tensile strain ratio of ≈10.2, while PVA/CS sample presented low fracture stress of only 0.2 MPa and <4.2 fracture strain ratio. Correspondingly, the fracture toughness (Γ) of samples was calculated. The Γ of the PVA/CS was only 5.30 kJ m^−2^, while that of H_PVA/CS_ can achieve as high as 26.02 kJ m^−2^. Figure 3i,j showed the snapshots of the PVA/CS and H_PVA/CS_ in during the real‐time fracture testing process. The crack of the PVA/CS expanded rapidly and then completely broke in less than 10 s, while the crack of H_PVA/CS_ expanded slowly. The mechanism responsible for this was that nanocrystalline domains within H_PVA/CS_ could protect the network from crack propagation. This meant that the H_PVA/CS_ could effectively inhibit the microscopic defects induced by fracture during deformation.

### 2.4 Characterizations of Layered Cartilage Hydrogels (H_PVA/CS‐PSPMA_)

Using multiple physically crosslinking strategy to improve the density of the polymer networks indeed could enhance the mechanical strength of bulk hydrogels, but it would weaken the surface hydration degree to induce poor lubrication ability. In nature, the collagen matrix in cartilage layer gives it good load‐bearing capacity while the hydrated bottle‐brush polysaccharide macromolecules on the superficial layer can maintain its lasting lubricity. This layered strategy inspired us to modify the surface of mechanically robust hydrogels for achieving good lubrication. Grafting hydrophilic PSPMA polyelectrolyte brush chains on the surface of the H_PVA/CS_ matrix with excellent load‐bearing and mechanical recovery properties could vividly simulate the biochemical structure of cartilage, for which would generate extraordinary lubrication performance.

**Figure 4:**
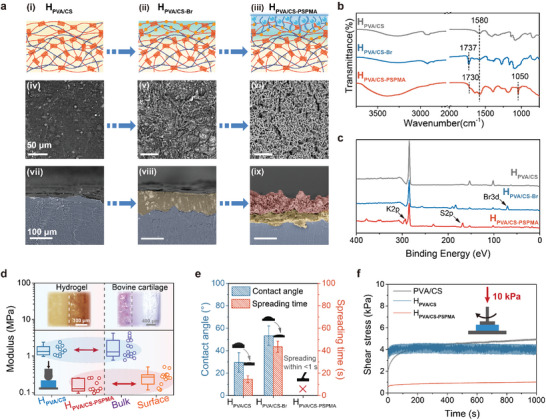
Characterizations of components, morphologies, and hydration states. a) Schematic diagram, surface images (i, ii, iii), and cross‐section SEM images (iv, v, vi) of H_PVA/CS_, H_PVA/CS‐Br_, and H_PVA/CS‐PSPMA_. b)The surface ATR‐FTIR spectra of H_PVA/CS,_ H_PVA/CS‐Br_, and H_PVA/CS‐PSPMA_. c) The surface XPS spectra of H_PVA/CS_, H_PVA/CS‐Br_, and H_PVA/CS‐PSPMA_. d) The surface moduli of the H_PVA/CS‐PSPMA_ and natural bovine articular cartilage are in an equilibrium swelling state. e) Water contact angles on the surfaces of H_PVA/CS,_ H_PVA/CS‐Br,_ and H_PVA/CS‐PSPMA_. f) The surface shear force of different hydrogels under 10 N load. Data in (E) are means ± SD, n = 3.

As shown in **Figure** **4**a and Figure S8 (Supporting Information), the morphologies of H_PVA/CS_ and H_PVA/CS‐PSPMA_ were characterized by Scanning Electron Microscope (SEM) and Energy Dispersive Spectrometer (EDS). The surface of H_PVA/CS_ was dense and flat (Figure 4a(iv)), while it became rough after anchoring the ATRP initiator layer (Figure 4a(v)), and generated much of the Br signals (Figure S9, Supporting Information). The surface of H_PVA/CS‐PSPMA_ appeared a large number of uniform micro‐pores after grafting PSPMA polyelectrolyte brushes (PB) chains (Figure 4a(vi)), and these micro‐pores would facilitate the considerable absorption of water molecules for providing rich surface hydration. As can be seen from the cross‐section images, the thickness of the ATRP initiator layer (H_PVA/CS‐Br_) on the surface of the H_PVA/CS_ matrix was 50–75 µm, while the thickness of PSPMA chains‐embedded composite lubrication layer on the surface of the H_PVA/CS‐Br_ was 60–80 µm (Figure 4a(vii,ix)). The hydrated and lubricating PB chains on the surface were covalently entangled with the sub‐surface of the nanocrystalline domain‐enhanced H_PVA/CS_ matrix, resulting in a continuous transition from the hydrated surface layer to the underlying bulk matrix. Correspondingly, a typical layered structure of H_PVA/CS‐PSPMA_ was obtained successfully. In addition, the successful preparation of H_PVA/CS‐PSPMA_ was confirmed by X‐ray Photoelectron Spectroscopy (XPS) and FT‐IR. Compared to the control H_PVA/CS_ sample (Figure 4b,c), the appearance of carbonyl (‐C═O) vibration peak at 1737 cm^−1^ (Figure 4b) and Br 3d signal peak at 70 eV (Figure 4c) on the surface of H_PVA/CS‐Br_, indicated the successful modification of the ATRP initiator. Furthermore, the appearance of carbonyl (‐C═O) vibration peak at 1730 cm^−1^ and characteristic absorption peak of the sulfonic group (S═O) at 1050 cm^−1^ (Figure 4b), as well as the generation of K2p and S2p signal peaks at 293 and 168 eV (Figure 4c), all well confirmed the successful formation of top composite lubrication layer after grafting PB chains.

Then, the surface and bulk moduli of H_PVA/CS‐PSPMA_ were measured by employing a soft matter nano‐indentation instrument, and compared them with the natural cartilage layer. The results showed that our strategy of bonding a soft PB layer onto a high‐strength H_PVA/CS_ matrix well simulates the mechanics of natural cartilage (Figure 4d). The elastic modulus of the surface PB layer was ≈0.16 MPa, while that of the bottom H_PVA/CS_ matix was 1.45 MPa, which was similar to the values of natural bovine articular cartilage (≈0.26 and 1.89 MPa). Correspondingly, grafting PSPMA on the surface of H_PVA/CS_ resulted in a significant wettability change from a hydrophilic state to super‐hydrophilic state (Figure 4e). The initial contact angles of water droplets were 29.5±8.76° on the surface of H_PVA/CS_ and 53.3±8.66° on the surface of H_PVA/CS‐Br_. By contrast, the surface of H_PVA/CS‐PSPMA_ was in a typical super‐hydrophilic state and the spreading time was <1 s, indicating the extraordinary surface hydration capacity of the top PB layer. This hydration capacity would enable its good swelling and rehydration feature under dynamic loading/shearing condition, which was beneficial to achieve durable and reliable lubrication. As validation, the shear stress on the surface of different samples was measured by employing a rheometer under small stress (10 kPa) (Figure 4f). It was found that the initial shear force on the surface of PVA/CS sample was very low, but which increased gradually along with time‐dominated dehydration. In contrast, the robust H_PVA/CS_ matrix showed high shear stress due to the poor surface hydration, while the surface of H_PVA/CS‐PSPMA_ achieved persistent and low surface shear stress because of its strong surface hydration ability.

### 2.5 Evaluation of the Lubrication Performance of H_PVA/CS_‐_PSPMA_

**Figure 5:**
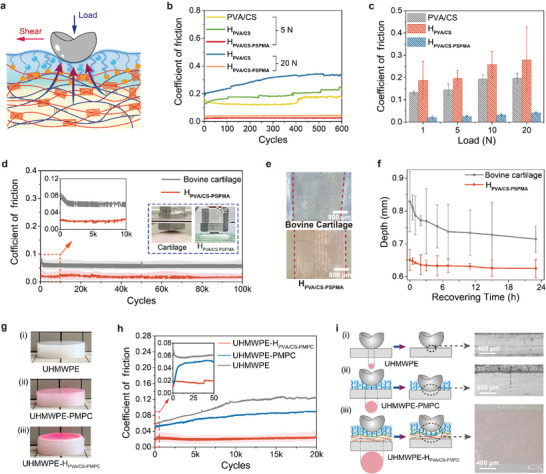
Lubrication, the anti‐wear performance of hydrogels, and corresponding demonstrations. a) Schematic diagram to show the friction shearing test process for H_PVA/CS‐PSPMA_ on a ball‐on‐disk contact mode. b) Representative COF curves and c) average COFs values of different hydrogels (frequency: 1 Hz, lubricant: PBS). d) The COF curves of H_PVA/CS‐PSPMA_ and natural bovine articular cartilage during the 100k sliding cycles (Lubricant: PBS, the normal load: 5 N, the frequency: 1 Hz). Fresh natural bovine articular cartilage was commercially purchased from a local market without treatment. e) Surface morphologies of the natural bovine cartilage and H_PVA/CS‐PSPMA_ after encountering 100k sliding cycles. f) The recovery process of deformation depth for bovine cartilage and H_PVA/CS‐PSPMA_ at sliding test area after 100k test cycles. g) Photographs of the blank UHMWPE, lubrication coating‐modified UHMWPE (UHMWPE‐PMPC) prepared by traditional photo‐initiated surface grafting and UHMWPE‐H_PVA/CS‐PMPC_ in this work. h) The real‐time COF curves of UHMWPE, UHMWPE‐PMPC, and UHMWPE‐H_PVA/CS‐PMPC_ during the 20k sliding cycles (Lubricant: PBS, the normal load: 5 N, the frequency: 1 Hz). i) Schematic diagram to show the local interface contact stress states at the friction shearing process for the UHMWPE‐PMPC (stiff contact) and UHMWPE‐H_PVA/CS‐PMPC_ (stress dissipation) on the ball‐on‐disk contact mode, and surface morphologies of test areas for UHMWPE, UHMWPE‐PMPC, and UHMWPE‐H_PVA/CS‐PMPC_ after completing 20k sliding cycles. Data in (C) and (F) are means ± SD, n = 3.

The lubricity of H_PVA/CS‐PSPMA_ was investigated systematically on a reciprocating ball‐on‐disk tribometer, where 316L steel balls with 6 mm diameter were used as friction pairs. To better simulate lubrication performances in the physiological environment, all friction tests were carried out in PBS buffer solution (pH 7.4) after achieving swelling equilibrium. Through the sphere‐ball friction contact mode, the influence of the load‐bearing capacity and surface lubrication of the sample on the COF of the whole system were investigated. The excellent load‐bearing capacity of H_PVA/CS_ enabled to achieve low COF by resisting mechanical deformation (*F_def_*), while the strong hydration of PB layer reduced the COF by classical hydration lubrication mechanism for minimizing the interface contribution (*F_int_*). By increasing the load‐bearing capacity of the matrix and improving the hydration degree of the surface based on the classical friction model, *F = F_int_+ F_def_*, the interface COFs can be significantly reduced (**Figure** **5**a). The specific friction comparison data were shown in Figure 5b. The poor bearing capacity of PVA/CS hydrogels caused a large mechanical deformation in shearing process as well as a high COF, while it generated wear easily under a low normal load of 5 N. Although the H_PVA/CS_ can resist mechanical deformation and present strong load‐bearing capacity of 20 N, the low surface hydration also led to a high COF. In contrast, H_PVA/CS‐PSPMA_ was able to maintain a stable and low COF under a 20 N load.

The COFs under different normal loads were also investigated. The COFs of H_PVA/CS‐PSPMA_ were always low and stable in a wide range of normal loads (Figure 5c). Correspondingly, the interface average contact stresses of the samples at different normal loads were calculated. The local contact stress between H_PVA/CS_ and steel ball increased obviously with the rising of normal loads from 1, 5, 10 to 20 N (5.51–7.90 MPa), while it decreased significantly when sliding against H_PVA/CS‐PSPMA_ (1.13–4.37 MPa) (Figure S10, Supporting Information). Therefore, the existence of the soft composite lubrication layer can effectively dissipate the contact stress under the same load condition. The interface sliding frequencies may also affect the lubrication behavior of hydrogels. The COFs of H_PVA/CS‐PSPMA_ increased gradually by extending the sliding frequencies from 0.2, 0.4, 1.0, 2.0 to 5.0 Hz, while that of the H_PVA/CS_ remained almost constant below 5 Hz (Figure S11, Supporting Information). The mechanism responsible for this can be explained by noticing that the composite lubrication layer was affected by the speed at which lubricant replenishes the contact. At higher speeds, a different equilibrium of fluid flow was reached corresponding to less lubricant being available for weeping into the contact. Furthermore, it was found H_PVA/CS‐PSPMA_ demonstrated good lubricity in different physiological media (Figure S12, Supporting Information).

Finally, the lubrication persistence of the H_PVA/CS‐PSPMA_ was tested by applying long‐period friction experiment (100k cycles) at a normal load of 5 N (2.06 MPa) with PBS as lubricant, while the fresh bovine cartilage was used for comparison. As shown in Figure 5d, the COF of the H_PVA/CS‐PSPMA_ was extremely stable and remained at ≈0.027 during the entire testing period. By contrast, even though the natural bovine cartilage also exhibited a stable lubrication state, the COF was higher than that of H_PVA/CS‐PSPMA_. Subsequently, the surface morphologies of the samples after friction tests were evaluated. Compared to morphologies before testing, slight surface scars at the sliding area were both observed for natural bovine cartilage and H_PVA/CS‐PSPMA_ after encountering 100k sliding cycles (Figure 5e). Meanwhile, the cumulative width of the wear scar of the H_PVA/CS‐PSPMA_ sample (≈2500 µm) was smaller than that of natural bovine cartilage (≈3200 µm), implying its stress‐dissipation capability to increase the degree of conformity with the rigid indenter surface. However, after encountering a long‐period friction test (100k cycles), it was observed that both surfaces at the sliding contact area deformed heavily. The deformation depth of natural bovine cartilage was 0.83 mm while that of H_PVA/CS‐PSPMA_ was only 0.65 mm (Figure 5f). Due to the good creep recovery property of H_PVA/CS‐PSPMA_, the deformation depth could recover to ≈0.62 mm after undergoing <24 h free recovery, while that of natural bovine cartilage was still as high as ≈0.72 mm. Even though H_PVA/CS‐PSPMA_ appeared to surface wear scars and mechanical deformation, its load‐bearing and lubricity were still better than that of natural bovine cartilage. These results indicate the excellent lubrication persistence, robustness, wear‐resistance, and mechanical recovery feature of H_PVA/CS‐PSPMA_ are comparable to but beyond the natural cartilage.

Furthermore, as proof of application concept in cartilage replacement on implant device, H_PVA/CS‐PSPMA_ material was tried to be used as lubrication coating on the surface of traditional UHMWPE, and then its lubricity was compared with the reported lubricating system that is surface‐grafted poly(2‐methacryloyloxyethyl phosphorylcholine) (PMPC) polyelectrolyte brushes coating.^[^ ^20^ ^]^ To achieve this purpose, H_PVA/CS‐PMPC_ was successfully prepared by grafting PMPC polyelectrolyte brushes onto the sub‐surface of the mechanically robust hydrogel (H_PVA/CS_) matrix.^[^ ^21^ ^]^ Then, H_PVA/CS‐PMPC_ was decorated onto the surface of UHMWPE as lubrication coating by applying a commercial biological adhesive as the bonding layer,^[^ ^10a^ ^]^ and UHMWPE‐H_PVA/CS‐PMPC_ was obtained successfully (Figure 5g). Correspondingly, the UHMWPE‐PMPC sample was also prepared by traditional photo‐initiated grafting polymerization technique.^[^ ^22^ ^]^ In order to evaluate the lubrication performances of UHMWPE‐H_PVA/CS‐PMPC_ and UHMWPE‐PMPC, the friction tests were investigated on a reciprocating ball‐on‐disk tribometer, where 316L steel balls with 6 mm diameter were also used as friction pairs and PBS buffer solution (pH 7.4) was used as lubricant. Compared to UHMWPE‐PMPC, UHMWPE‐H_PVA/CS‐PMPC_ showed lower COFs in a wide range of normal loads (1, 2, 5, 10, 20 N) (Figure S13, Supporting Information). This may be attributed to the grafting density and thickness (≈dozens of microns) of hydrated PMPC polyelectrolyte brush chains on the surface of UHMWPE‐H_PVA/CS‐PMPC_ are obviously higher than that of UHMWPE‐PMPC (≈dozens of nanometer). The lubrication persistence of bare UHMWPE, UHMWPE‐PMPC, and UHMWPE‐H_PVA/CS‐PMPC_ were further evaluated by applying a long‐period friction experiment (20k cycles, 5 N) in PBS. The COF of bare UHMWPE increased continuously from 0.06 to 0.12 within 15 000 cycles and then kept stable, resulting from poor surface hydration lubrication. For UHMWPE‐PMPC, the sliding interface presented an ultralow COF (≈0.01) at the start‐up stage, but soon rose to a relatively high level (COF: ≈0.04) in the 20th second, and then gradually increased with extending the sliding cycles. When the sliding cycles reached 20k, the surface COF of UHMWPE‐PMPC increased to 0.09. By contrast, the COF of UHMWPE‐H_PVA/CS‐PMPC_ always remained stable and low (COF: ≈0.028) during the entire sliding period (Figure 5h).

Subsequently, the surface wear states of these three kinds of samples were investigated by observing the morphologies of the sliding test areas. The wear scar width was ≈523 µm for bare UHMWPE (Figure 5i(i)), while it increased to ≈664 µm for UHMWPE‐PMPC (Figure 5i(ii)). As a stark contrast, the wear scar width of UHMWPE‐H_PVA/CS‐PMPC_ was as large as ≈1435 µm (Figure 5i(iii)). Under the same loading and testing conditions, the difference in wear scar width well reflects the contact stress situation at the sliding interface. The local interface contact stress of bare UHMWPE was as high as 47.80 MPa, while it decreased slightly after grafting PMPC brushes chains as the lubrication layer (25.74 MPa). In this case, due to the thin thickness of the PMPC brushes, the interface was still in a typical stiff contact state, so the lubrication layer was easily worn out. By comparison, our layered H_PVA/CS‐PMPC_ coating on the surface of UHMWPE could not only provide lubrication but also effectively dissipate the interface contact stress (5.55 MPa). As a robust/low‐friction coating, H_PVA/CS‐PMPC_ exhibited stronger load‐bearing capacity and more lasting lubricity than that of traditional surface‐grafted PMPC polyelectrolyte brushes coating.

### 2.6 Evaluation of the Cytocompatibility and Anti‐Proteins Property of H_PVA/CS‐PSPMA_

The Cell Counting Kit‐8 (CCK‐8) cell viability assays were carried out to investigate the cytotoxicity of H_PVA/CS‐PSPMA_. Specifically, mouse embryonic osteoblast precursor cells (MC3T3‐E1) (Density: 1×10^4^ cells/well) were cultured on the surface of H_PVA/CS‐PSPMA_ for 1, 3, and 5 days in MEM‐α medium, while the same cells on TCP plates were used as a control group. Correspondingly, 100 µL of complete medium containing 10% CCK‐8 was added into the medium to incubate for 2 h, and then the absorbance was recorded at 450 nm in a Microplate Reader. The experiments were repeated at least three times. As shown in Figure S14 (Supporting Information), the cell viability remained all above 80% for 1, 3, and 5 days, indicating the low toxicity of the H_PVA/CS‐PSPMA_. Furthermore, the anti‐protein capacity is also an essential performance for implant material. After immersing into fluorescein isothiocyanate labeled bovine serum proteins (FITC‐BSA) (0.5 mg mL^−1^) for 3 h, the samples were taken out for fluorescence imaging under a confocal laser scanning microscope (CLSM). Compared to the strong BSA proteins attachment of the control sample without the composite lubrication layer (H_PVA/CS_), the protein adhesion on the surface of H_PVA/CS‐PSPMA_ decreased significantly (Figure S15, Supporting Information). The fluorescence intensities on each image were analyzed by the ImageJ software (NIH, USA). The mean fluorescence intensities of H_PVA/CS‐PSPMA_ decreased to ∼13.8%, relative to that of H_PVA/CS_ is 67.9% (Figure S16, Supporting Information). This could be well explained as the thick surface hydration layer of H_PVA/CS‐PSPMA_ could effectively resist the approach of protein molecules. Combined with low cytotoxicity and good anti‐protein properties, our H_PVA/CS‐PSPMA_ presents great application potential as cartilage replacement or implant coatings in vivo.

## 3 Conclusion

Natural cartilage as can achieve ultra‐long lubricity lifetime at physiological media under high contact pressure condition, developing mechanically robust artificial cartilage materials is still challenging. In this work, we engineered one kind of novel cartilage‐like hydrogels (H_PVA/CS‐PSPMA_) with high load‐bearing, low‐friction, wear‐resistance, and deformation recovery properties by covalently manufacturing thick composite lubrication layer through the sub‐surface of the mechanically robust hydrogels matix (H_PVA/CS_). The H_PVA/CS‐PSPMA_ exhibited high mechanical strength (compression modulus: 11.8 MPa, compression strength: 15.25 MPa), good anti‐swelling ability in the physiological medium, and typical creep recovery characteristics. The H_PVA/CS‐PSPMA_ could present good lubricity (COF: 0.02–0.03) in PBS under high contact pressure condition (2.0–3.0 MPa). Surprisingly, under harsh friction test condition (100k cycles, *P* ≈2.5 MPa), the H_PVA/CS‐PSPMA_ could maintain lower and more stable COF (≈0.027) than that of natural bovine cartilage, as well as ignorable surface wear. More importantly, the mechanical deformation at the sliding test area of H_PVA/CS‐PSPMA_ could recover considerably after the friction test. The excellent lubrication persistence, robustness, wear‐resistance, and mechanical recovery feature of H_PVA/CS‐PSPMA_ are comparable to but beyond the natural animal cartilage. Finally, as proof of concept, H_PVA/CS‐PMPC_ was prepared and then decorated onto the surface of implantable UHMWPE as a robust/low‐friction coating, which exhibited higher load‐bearing capacity and more lasting lubricity than that of traditional surface‐grafted PMPC polyelectrolyte brushes coating. However, the experiments conducted in current research were in vitro friction tests, further validation of coating application value requires big animal experiments and long‐term clinical trials, which is still a long way to go. The basic design concept of this work provides a new route for developing wear‐resistant cartilage replacement and artificial joint coatings.

## Conflict of Interest

The authors declare no conflict of interest.

## Author Contributions

S.M. and F.Z. conceived the idea. S.M. and W.Z. designed the experimental protocol. W.Z. and Y.Z. performed and completed the entire experimental study. X.Z. provided technical suggestions. S.M. and W.Z. wrote the paper. W.S. helped to modify language and grammar. S.M. and F.Z. revised and finalized the manuscript. The authors also thank to kind assistance from Y. Hu in LICP to perform the anti‐fouling experiment.

## Supporting information

Supporting Information

## Data Availability

The data that support the findings of this study are available from the corresponding author upon reasonable request.
