# Supplementary material for: Mechanically Robust Lubricating Hydrogels Beyond the Natural Cartilage as Compliant Artificial Joint Coating
Source: Adv Sci (Weinh). 2024 Jun 17;11(31):2401000. doi: 10.1002/advs.202401000 (PMC11336983; doi:10.1002/advs.202401000)
Supplement: Supplementary file 1 — Supporting Information [file ADVS-11-2401000-s001.docx]

Supplemental Information

**Mechanically Robust Lubricating Hydrogels Beyond the Natural Cartilage as Durable Artificial Joint Coating**

*Weiyi Zhao, Yunlei Zhang, Xiaoduo Zhao, Wenbo Sheng, Shuanhong Ma*, Feng Zhou**

Dr. W. Zhao, Y. Zhang, Dr. X Zhao, Prof. W Sheng, Prof. S. Ma, Prof. F. Zhou

State Key Laboratory of Solid Lubrication

Lanzhou Institute of Chemical Physics

Chinese Academy of Sciences

Lanzhou 730000, China

E-mail: mashuanhong@licp.cas.cn; zhouf@licp.cas.cn

Dr. W. Zhao

Center of Materials Science and Optoelectronics Engineering, University of Chinese Academy of Sciences, Beijing, 100049, China

Dr. X Zhao, Prof. S. Ma

Shandong Laboratory of Advanced Materials and Green Manufacture at Yantai, Yantai Zhongke Research Institute of Advanced Materials and Green Chemical Engineering, Yantai, 264006, China

**Supplemental Note 1. Experimental Section**

**Materials:**

Poly (vinyl alcohol) (PVA) (1750) and Na_3_Cit were purchased from Sinopharm Chemical Reagent Co., Ltd. Short-chain chitosan (CS) (degree of deacetylation >90%, viscosity 45 MPa s for 1% (w/v), and molecular weight: ~10 kPa) was purchased from Jinhu Company, China. 2-bromoisobutyryl bromide (98%) was purchased from J&K Chemical Ltd. and used as received.2,2’-bipyridine (Bipy, 99%), copper (Ι) bromide (CuBr), and 3-sulfopropyl methacrylate potassium (SPMA) were purchased from TCI Co., Ltd. CuBr was purified by stirring overnight in acetic acid. N-methyl-2-pyrrolidone (NMP) (99%) was purchased from Tianjin Kemiou Chemical Reagent Co., Ltd. 2-Methacryloyloxy ethyl phosphorylcholine(MPC) (96%) was purchased from Shanghai Macklin Biochemical Technology Co., Ltd. Methanol (99.9%) was purchased from Beijing Merida Technology Co., Ltd. RelyX™ Ultimate was purchased from3M company (Minnesota Mining and Manufacturing Company). Deionized (DI) water was purified in the laboratory. All chemical agents were directly used without any purification. Fresh natural bovine articular cartilage were commercially purchased from a local market without treatment.

**Synthesis of composite hydrogels:**

PVA (12.5 wt %) was dissolved into DI water at 100 °C with vigorous magnetic stirring and then cooled to RT. CS powder (12.75 wt %) was added to the PVA solution with mechanical stirring. The as-prepared hydrogel solution was kept overnight at room temperature (RT) to ensure complete hydration. The samples were subjected to three freeze-thaw cycles where they were frozen at -16°C for 8 h and then thawed at room temperature for 6 h. The prepared samples were then immersed in a 1.3 M Na_3_Cit solution for 24 h and then in DI water for 24 h to remove excess Na_3_Cit. Finally, the samples were annealed in an oven at 100°C for 90 min.

Surface grafting of anionic polymer brushes: ATRP initiator functionalization was accomplished by immersing each annealed sample in NMP containing 0.4 M 2-bromoisobutyryl bromide at 50 °C for 1 h. The SPMA monomer (6 g) was dissolved in the 12 mL water/methanol (the ratio is 2:1) at RT and degassed for 30 min with N_2_. And then, the Bipy (80 mg) and CuBr (35 mg) were added to this solution in the Schlenk tube successively. The mixture was further stirred and degassed with N_2_ for another 20 min. The samples were put into the reaction solution under N_2_ protection without being stirred for 1 h. Finally, the samples were taken out and washed with DI water to remove any unreacted monomers and catalysts.

**Preparation of UHMWPE-PMPC:**

After the UHMWPE block (d=30mm，h=10mm) was washed twice with methanol using an ultrasonicator for 30 min, the block was immersed in acetone solution containing benzophenone (1.0 g/dL) for 30 s. The sample was dried under dark condition for 1 h in vacuo. The MPC solution was prepared using degassed pure water. The concentration of the MPC was 0.5 mol/L. A volume 30 mL of the MPC solution was placed in a glass tube and argon gas was passed through the solution for 3 min to eliminate any oxygen. The UHMWPE block coated with benzophenone was placed in this tube and the glass tube was then sealed. The photo-polymerization on the UHMWPE surface was carried out using a 365 nm UV lamp at 60 °C ^[1]^.

**Preparation of UHMWPE-H_PVA/CS-PMPC_:**

The H_PVA/CS_ sheet (thickness: 1 mm) after salting out and annealing is fixed to the surface of the UHMWPE block ^[2]^. Scotchbond Universal Adhesive was applied to the layer of the H_PVA/CS_ in contact with the UHMWPE bulk and the top surface of the bulk. The adhesive was allowed to set for 20 seconds before being blown by air for another 5 seconds. About 0.15 g of RelyX™ Ultimate Cement was then applied to the same surfaces coated with the Scotchbond Universal Adhesive. H_PVA/CS_ and UHMWPE are bonded together and then pressed into a custom grinder to hold. The cement was cured for 1 h, and the sample was then soaked in DI water for 1 hour in a centrifuge tube before future use. Sample after plasma treatment, CuBr (0.025 mmol), and Bipy (0.050 mmol) were introduced into a glass tube with a stopcock and were dried by repeating degas and argon purge. A methanol solution of MPC (6.5 mmol) was added to the catalyst. The polymerization reaction was conducted at 303 K for 12 h. The resulting sample is washed well with methanol and DI water to remove residual catalyst.

**Characterization of the morphology**

The SEM (JSM-5600LV at an accelerating voltage of 20 kV) and optical microscope Olympus BX51 were employed to observe the morphology of the samples. The samples for SEM tests were treated with liquid nitrogen for 10min and then dried at −40°C in 1 Pa for 24 h (SCIENTZ-10N, Ningbo Scientz Biotechnology Co., Ltd.). Static contact angle (CA) was measured using a DSA-100 optical contact angle meter (Krüss Company, Ltd., Germany) at ambient temperature (25 °C).

**Characterization of the components**

The elemental composition was evaluated using energy dispersive spectroscopy (EDS, attached to the SEM apparatus). The surface chemistry components of samples were also characterized by X-ray photoelectron spectroscopy (XPS) using a Thermo ESCLAB 250Xi spectrometer. Meanwhile, the C1s line at 284.6eV from adventitious carbon was used as a reference. The chemical compositions of samples were evaluated using attenuated total reflection-Fourier transform infrared spectroscopy (ATR-FTIR) on a Perkin-Elmer Transform Infrared Spectrometer (Perkin-Elmer, USA).

**SAXS and WAXS measurements**

To probe the crystal dimension, distance between crystalline domains, interlamellar spacing, as well as the change in the crystalline domains during the hydrogel fabrication, light scattering measurements were conducted. WAXS measurements were carried out using the Xeuss 2.0 (Xenocs, France), with a Cu-Kα radiation wavelength of 1.54060 Å. Two-dimensional (2D) SAXS were collected via an X-ray detector of HyPix-6000. The sample holder was mounted onto an optical table, and the sample-to-detector distance was set at 1185 mm for SAXS and 88 mm for WAXS.

The efficient scattering range of q was 0.002~0.820 nm^−1^ for the SAXS measurements, and the efficient diffraction angle range of 2θ was 0~50^◦^ for the WAXS measurements. The measured scattering intensity of the hydrogels at the swollen state was corrected by subtracting the water and air background. Quantification of SAXS and WAXS patterns was performed with scattering vector (*q*) and azimuthal angle (*2θ*) as coordinates. SAXS was used to characterize the inter-crystal spacing, and WAXS was utilized to characterize the crystal dimension, lattice spacing.

The inter-crystal spacing (*L_2_*) of material was quantified by the one-dimensional scattering curve of corrected scattering intensity (*Iq^2^*) versus scattering vector (*q*) obtained by 2D SAXS patterns, as follows:

$L_{2}=2\pi/q$(1),

$q=4\pi\left( sin\theta\right)/\lambda$(2),

where *λ* is the diffraction wavelength. The lattice spacing (*D*) of PVA material was quantified by the one-dimensional scattering curve of intensity versus diffraction angle (*2θ*) obtained by the ($10\bar{1}$) reflection in WAXS patterns, calculated using the following Scherrer’s equation:

$D=k\lambda/\beta\cos\theta$(3),

where *β* is the half width of the maximum diffraction peak, and *k* is the dimensionless shape factor. The lattice spacing (*L_1_*) was determined from the one-dimensional scattering curve of intensity versus scattering vector (*q*) obtained by 2D WAXS patterns, using equations (1) and (2).

**Measurement of crystallinity**

The crystallinities of all samples in their dry state were quantified by a Differential Scanning Calorimetry (DSC, STA449F3), following the previously-reported protocol. All the samples were first chemically cross-linked with excessively glutaraldehyde, in order to fix the amorphous polymer chains before air-drying, thus no further crystallization will occur during the drying process.

We measured the mass of residual water (*m_residual_*), the mass of crystalline domains (*m_crystalline_*), and the total mass of the dry samples with residual water (*m_total_*) by DSC test.

All the hydrogel samples were first air-dried under room temperature and the total mass was measured as *m_total_* before the DSC test. For the DSC test, the air-dried samples were heated up from 20 °C to 250 °C at a rate of 20 °C min^−1^, under a nitrogen flow of 30 mL/min. The mass of the residual water *m_residua_*_l_ can be calculated as$m_{residual}=m_{total}\times\frac{H_{residual}}{H_{water}^{0}}$, where $H_{water}^{0}=2260J/g$is the latent heat for water evaporation.

The enthalpy for melting the crystalline domains per mass unit of the dry sample (with residual water) *H_crystalline_* can be estimated by integrating the endothermic transition ranging from 200 °C to 250 °C. Therefore, the mass of the crystalline domains (*m_crystalline_*) can be calculated as $m_{crystalline}=m_{total}\times H_{crystalline}^{0}$, where $H_{crystalline}^{0}=138.6 J/g$is the enthalpy of melting of 100% crystalline PVA. Therefore, the crystallinity of dry samples *X_dry_* (without residual water) can be calculated as ^[3]^

$X_{dry}=\left( \frac{m_{crystalline}}{m_{total}-m_{residual}} \right)\times100\%$(4)

**Characterizations of mechanical properties**

The characterizations of the mechanical properties were performed on a General Electric material testing machine (EZ-Test, SHIMADZU) with a 500 N load cell. The tensiletests were performed at room temperature at tensile deformation rate of 100 mm/min. The dumbbell-shaped samples (2 mm (W)×13.5 mm (L)) were cut using a die cutter following the standard of ISO4661-1 in order to avoid systematic failure in the vicinity of the clamps. The compression speed was 5 mm/minofcompressiontests. The sample size was 10 mm×10 mm×2 mm, and silicone oil was applied to the surface of the hydrogel. The result obtained was the average of the three measured values for each sample.

The characterization of the surface modulus was performed on a Bioindenter (Anton Paar, UNHT^3^ Bio, Switzerland).

Young’s modulus was calculated at the slope range from 5 to 15% of the strain of stress−strain curves. The *Γ* of hydrogels, a parameter that characterizes the work required to fracture the sample per unit volume, was calculated with the corresponding tensile stress−strain curves. Specifically, the *Γ* can be calculated by the followed equation ^[4]^

$\Gamma=\int_{\varepsilon=0}^{\varepsilon=\varepsilon_{b}} \sigma_{load}d\varepsilon$ (5)

where *σ_load_* and *ε_b_* are the corresponding stress and breaking strain during the loading process, respectively.

The contact stress and deformation analysis were based on Hertz contact theory ^[5]^. Paraments in Hertz contact model were used from the experimental results in this work. Mechanical property paraments of hydrogels were obtained from related data in mechanical test. In order to simplify the analysis, we carried out a macroscopic indentation test (indentation radius (*R*): 3 mm, load (*F*):1-20 N). Displacement (*d*) of samples in macroscopic sphere indentation can be transformed into contact radius (*a*) by geometry condition in Hertz model.

$a^{2}=Rd$ (6)

And the contact stress (*P*) was calculated by:

$P=\frac{F}{\pi a^{2}}$(7)

The hydrogels were cut into rectangular specimens with a height of 40 mm and a width of 20 mm for the fracture tests. The thickness of individual specimens was measured with a calliper. An initial clamp distance of 1 mm or 2 mm was used for every pair of specimens. All specimens had microstructure alignment parallel to the height direction. For pure shear tests, two identical samples (one notched, one unnotched) were loaded under the sample setup as a pair to obtain one fracture energy value. Briefly, for the notched samples, an initial 8-mm-long straight cut was made from the middle of the long edge towards the center of the hydrogels, and the specimen was loaded at a strain rate of 10% s^−1^. The critical strain (ε_c_) for unstable propagation of the crack was obtained from the strain at maximum stress. The pairing unnotched specimens were subsequently loaded until ε = ε_c_. The fracture energy value was obtained by multiplying the area under the stress-strain curve of the unnotched specimens with the initial clamp distance (H) as ^[6]^,

$\Gamma=H\int_{0}^{\varepsilon_{c}} \sigma d\varepsilon$ (8)

**Evaluation of lubrication performance**

The lubrication performance of the samples was tested on conventional reciprocating tribology (CSM, TRB, Switzerland) instrument, and the coefficients of friction (COF) under different text conditions were recorded. Steel hemispheres (diameter: 6 mm) (ball-on-disk mode) were used as counterpairs, and deionized water was used as a lubricant. The distance of one sliding cycle was 10 mm. Each friction test was performed by sliding at a sliding speed of 0.01 m/s under an applied load. The result obtained was the average of the three measured values for each sample.

**Rheology behavior**

The shear resistances of hydrogels were measured by a rheometer (HAAKE, RS6000, Germany) with a coaxial two-parallel plate model, in which the clamp diameter was 35 mm. The storage modulus (G’) was measured at a frequency of 1 Hz and the shear stress changed from 0.01 to 10000 Pa. All the measurements were carried out at RT.

**X-ray diffraction (XRD)**

XRD patterns for samples were obtained on a diffractometer (Smartlab-SE, Rigaku Corporation, Japan) with Cu Kα radiation (1.542 nm) at 40 kV and 40 mA in the 2 theta range of 5–45 ° at room temperature.

**Swelling properties**

Swelling measurement was carried out to explore the swelling properties of hydrogels. The weighed samples were placed in excess PBS buffer solution(pH=7.4) at RT for a certain time. The immersing specimens were taken out for weighing again by draining the surface water at a fixed interval. The swelling ratio (*SR%*) can be estimated using the following equation, where *Wi* is the initial weight of hydrogels, and *Wt* is the wet weight of hydrogels at different intervals during the immersing process ^[7]^. The result obtained was the average of the three measured values for each sample.

$SR\%=\frac{W_{t}-W_{i}}{W_{i}}\times100\%.$ (9)

**Evaluation of biocompatibility**

CCK-8 assay to detect the effect of hydrogels on the proliferation of mouse embryonic osteoblast precursor cells (MC3T3-E1).

**Cell culture**

MC3T3-E1 cells were cultured using MEM-α medium (iCell, item no: iCell-0003). Medium: 90% MEM-α medium, 10% fetal bovine serum (iCell Bioscience Inc, item no.: iCell-002b) to prepare cell complete medium, 1% penicillin/streptomycin. Culture conditions: 5% CO_2_, 37°C constant temperature incubator.

**Sample pre-treatment**

The samples were cut into small slices of about 5mm×5mm×1mm, sterilized under UV light for 2 h and finally rinsed three times with phosphate buffer (Procell, item no. WH0112201 911XP) before being prepared for use.

**CCK-8 experiment**

MC3T3-E1 cells at logarithmic growth stage were counted, the cell concentration was adjusted and the cells were inoculated with a density of 1×10^4^ cells/well into 96-well plates containing hydrogel, 5% CO_2_ and incubated at 37°C in a constant temperature incubator; the cells were incubated for 1, 3 and 5 day; the medium containing the samples was removed and each well was incubated with 100 μL of complete medium containing 10% CCK-8 (Mei5 Biotechnology Co., Ltd., No. MF-128-02) at 5% CO_2_ for 2 h in a constant temperature incubator at 37°C. The absorbance of the cells was measured at 450 nm. The results were expressed as mean ± standard deviation. One-way ANOVA analysis was used to determine the statistically significant differences between the groups. p<0.05 was considered statistically significant.

**The nonspecific protein adsorption test**

The nonspecific protein adsorption test of choosing the Fluorescein isothiocyanate labeled bovine serum protein (FITC-BSA) bought from Beijing Solarbio Science & Technology Co. Ltd was performed. The specific procedure is as follows: Firstly, the H_PVA/CS_ samples and H_PVA/CS-PSPMA_ samples were cut into 1×1 cm^2^ dices and immersed into PBS buffer solution (pH=7.4) to remove the adsorbed impurity; then, these samples were dried with a filter paper and transferred into little vials along with the addition of the 2 mL FITC-BSA (0.2 mg/mL) solution; finally, the vials were kept for 3 h to adsorb. After adsorption, the samples were rinsed with fresh PBS buffer solution three times to remove the non-adhered protein. The adsorption behavior of proteins was characterized by a laser scanning confocal microscope (LSCM, Olympus, FV1200, magnification 10X).

**Supplemental Note 2. Supplemental experimental results (Supplemental Figure 1- Supplemental Figure 16)**


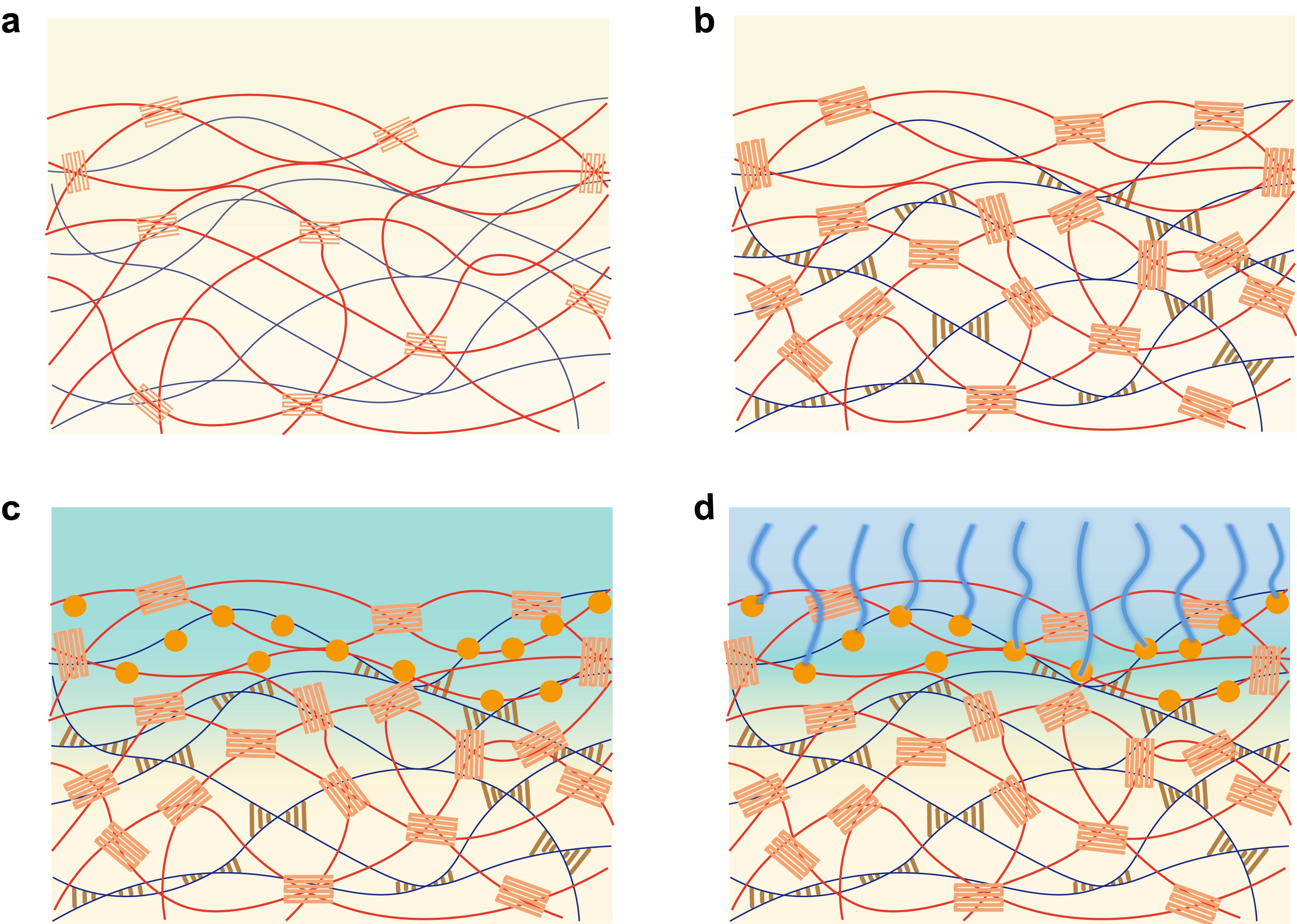


**Figure S1.** The preparation process of H_PVA/CS-PSPMA_. (a) Preparation of PVA/CS hydrogel by the freeze-thaw method. (b) Preparation of H_PVA/CS_ hydrogel by salting out and annealing. (c) Preparation of ATRP initiator-tethered H_PVA/CS_(H_PVA/CS-Br_) by surface chemistry reaction. (d) Preparation of lubricious H_PVA/CS-PSPMA_ by sub-surface grafting PSPMA polyelectrolyte brush chains.


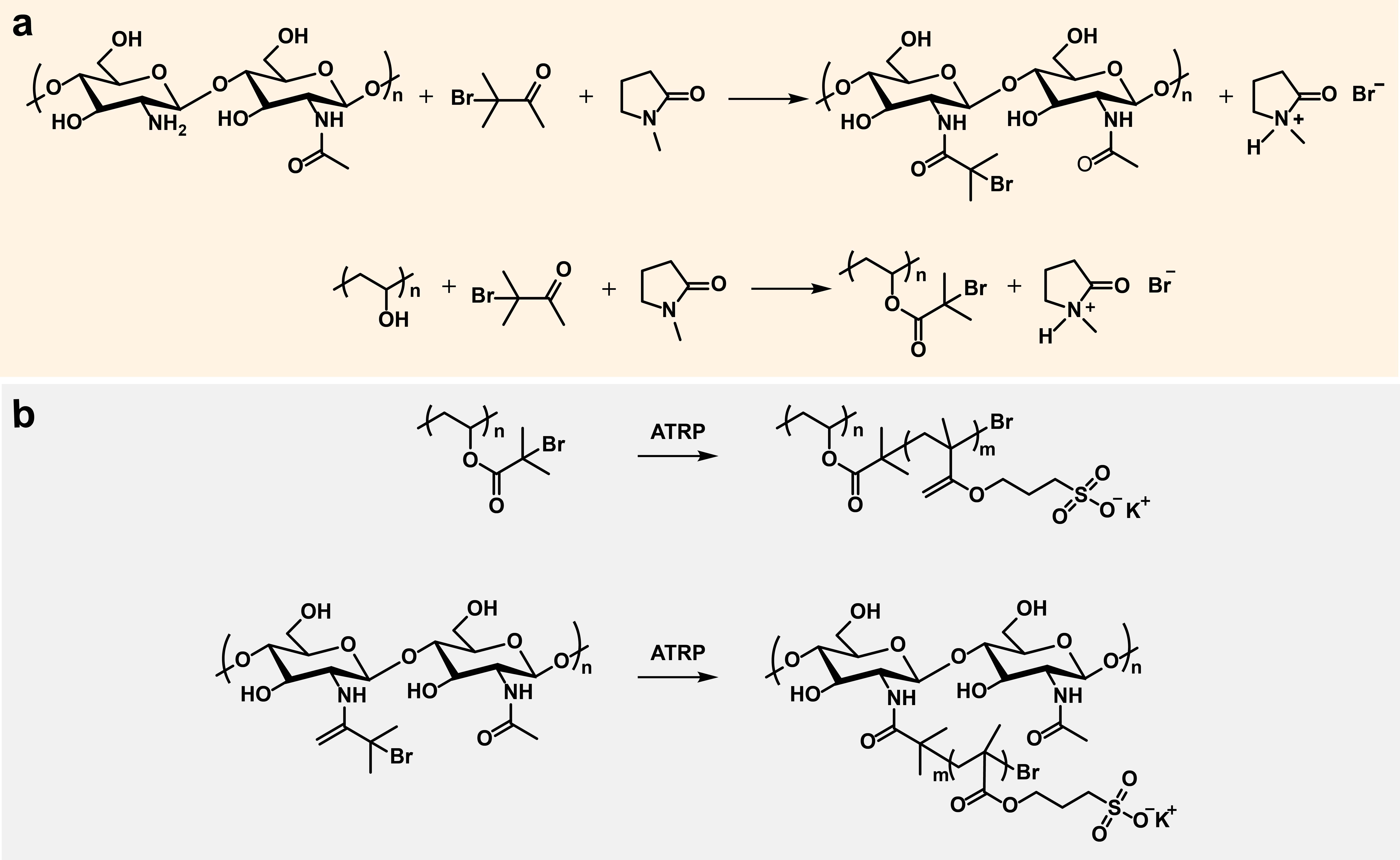


**Figure S2.** (a) Functionalization of ATRP initiator α-bromoisobutyryl bromide to the surface of a dehydrated H_PVA/CS_ matrix. (b) Grafting PSPMA brush to the H_PVA/CS-Br_ matrix.


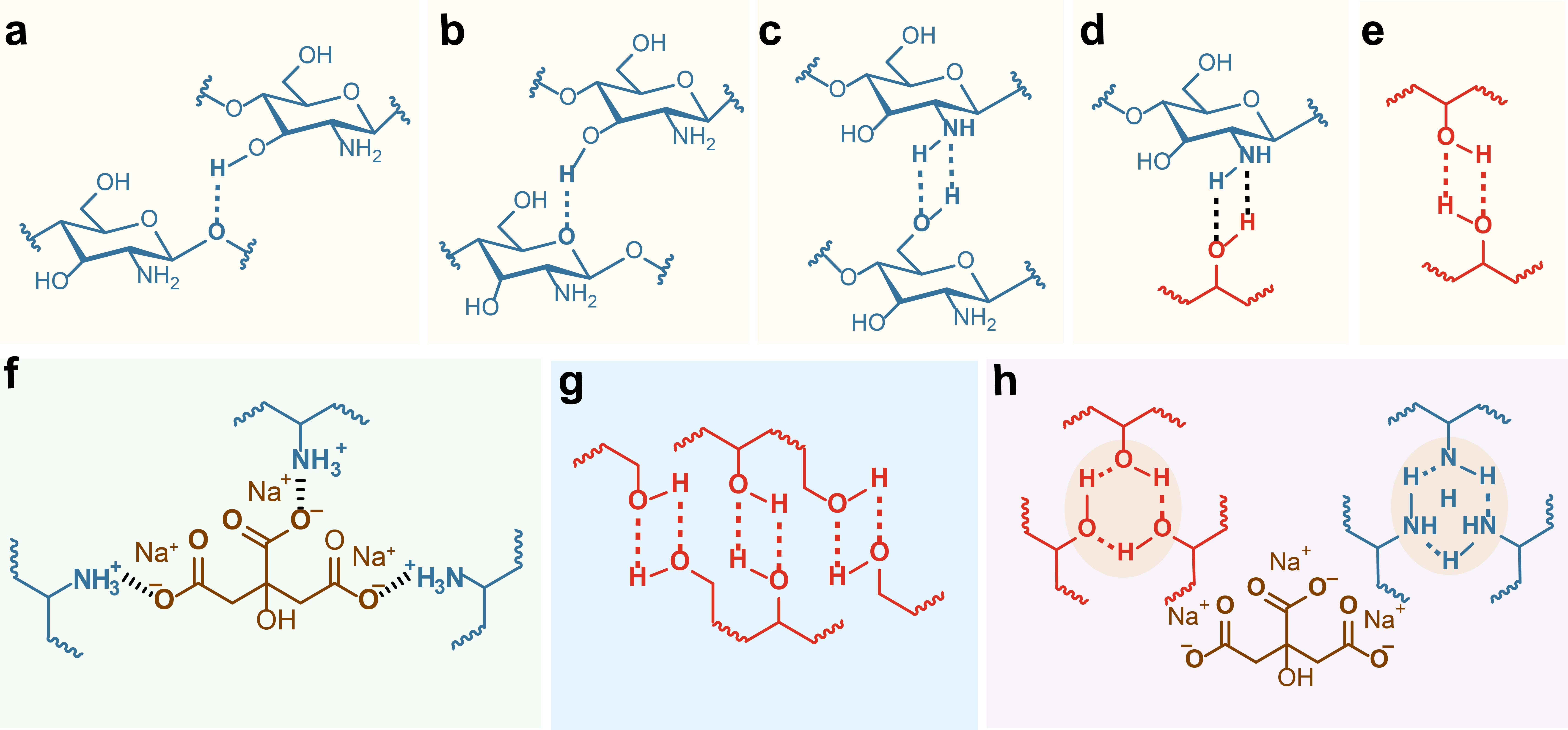


**Figure S3.** The dominant molecular interactions of H_PVA/CS_ matrix in the whole manufacturing steps. (a), (b) and (c) Molecular interactions of CS/CS. (d) Molecular interaction of PVA/CS. (e) Molecular interaction of PVA/PVA. (f) Coordination network of sodium citrate (Na_3_Cit)/CS. (g) Hydrogen bond network between chains of PVA molecules by freeze-thaw and annealing crystallization. (h) Molecular interactions of H_PVA/CS_ matrix.


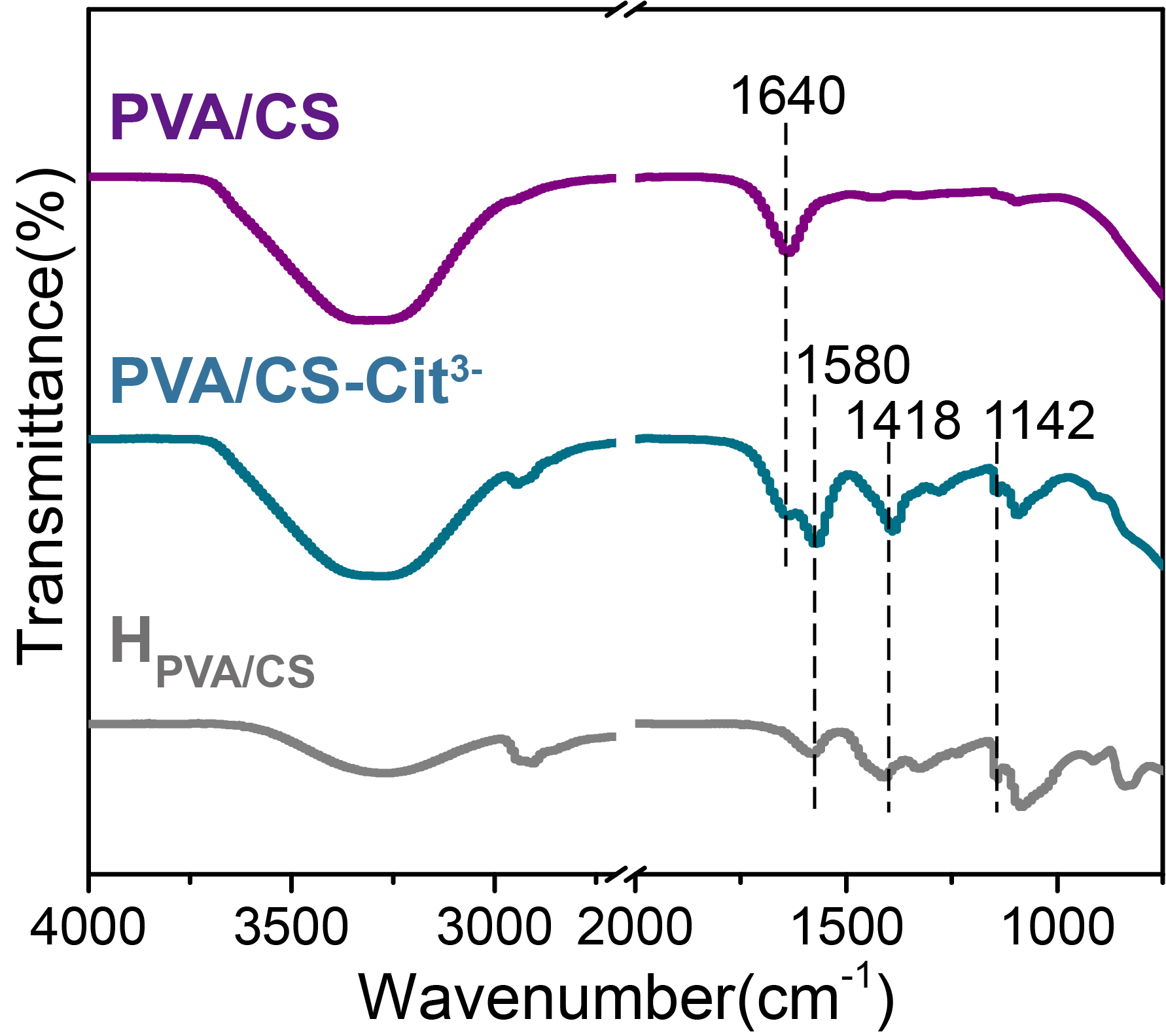


**Figure S4.** The surface ATR-FTIR spectra of the PVA/CS, PVA/CS-Cit^3-^ and H_PVA/CS_. (1640 cm^-1^: absorption peak of amide I; 1142 cm^-1^: characteristic peak of PVA crystallization; 1580 cm^-1^: Antisymmetric stretching vibration peak of -COO^-^; 1418 cm^-1^: symmetric stretching vibration peak of -COO^-^).


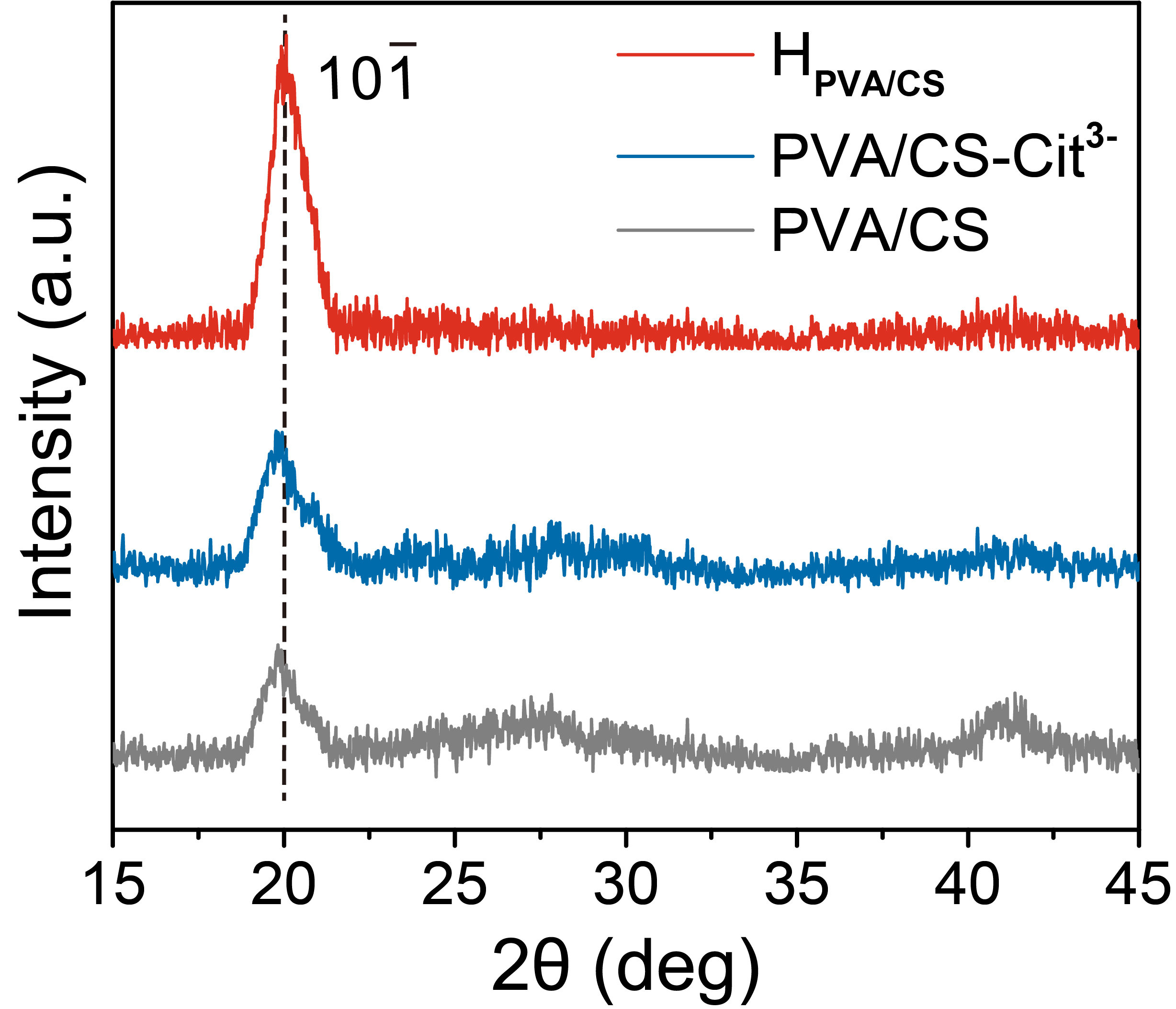


**Figure S5.** XRD spectra of the PVA/CS hydrogel (first hydrogen bond network: without salting out and annealing treatment), PVA/CS-Cit^3-^ hydrogel (first hydrogen bond network and second ions coordination network: without annealing treatment), and H_PVA/CS_ (first hydrogen bond network, second ions coordination network and third hydrogen bond network).


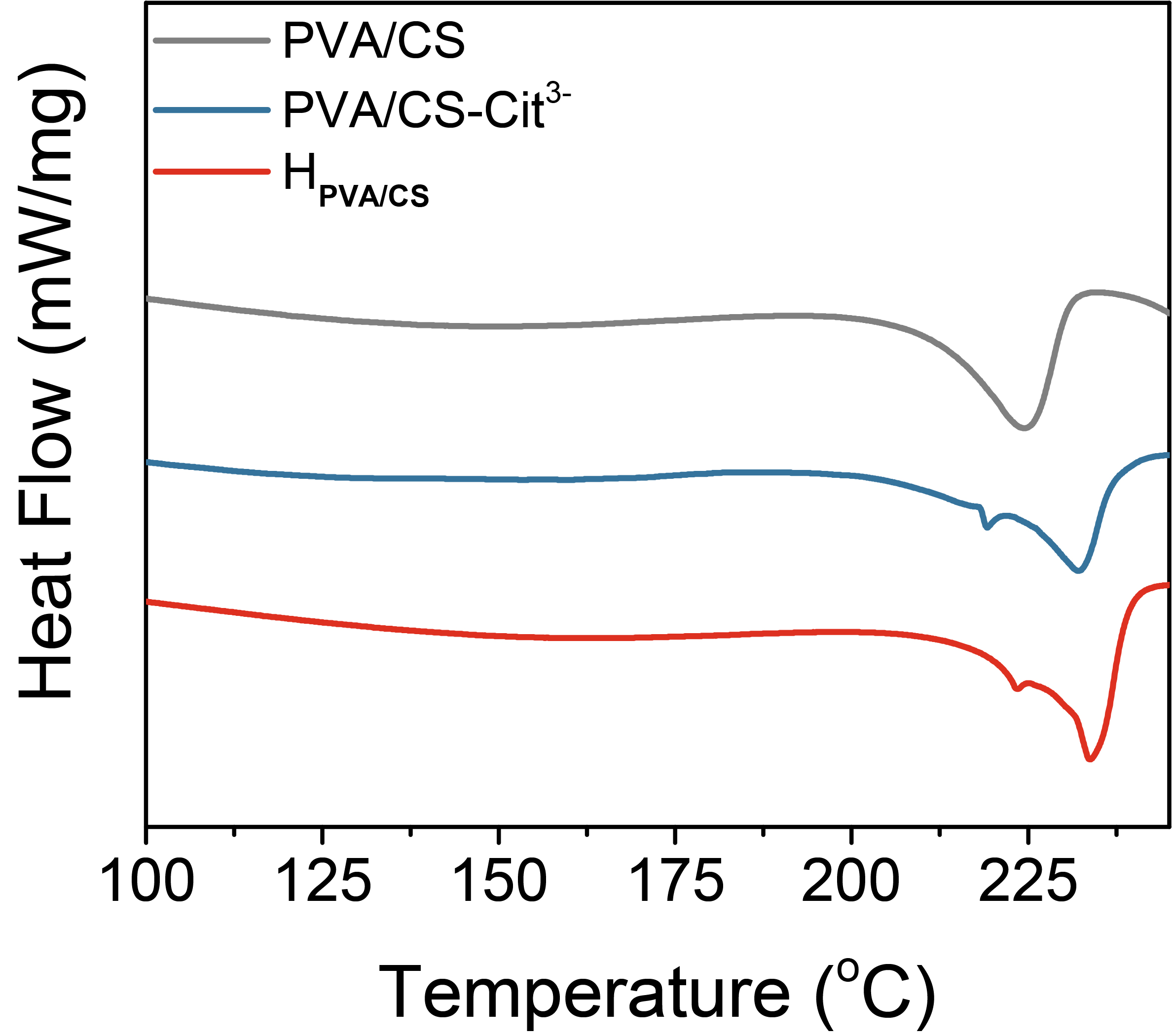


**Figure S6.** DSC measurement of crystallinity of samples.


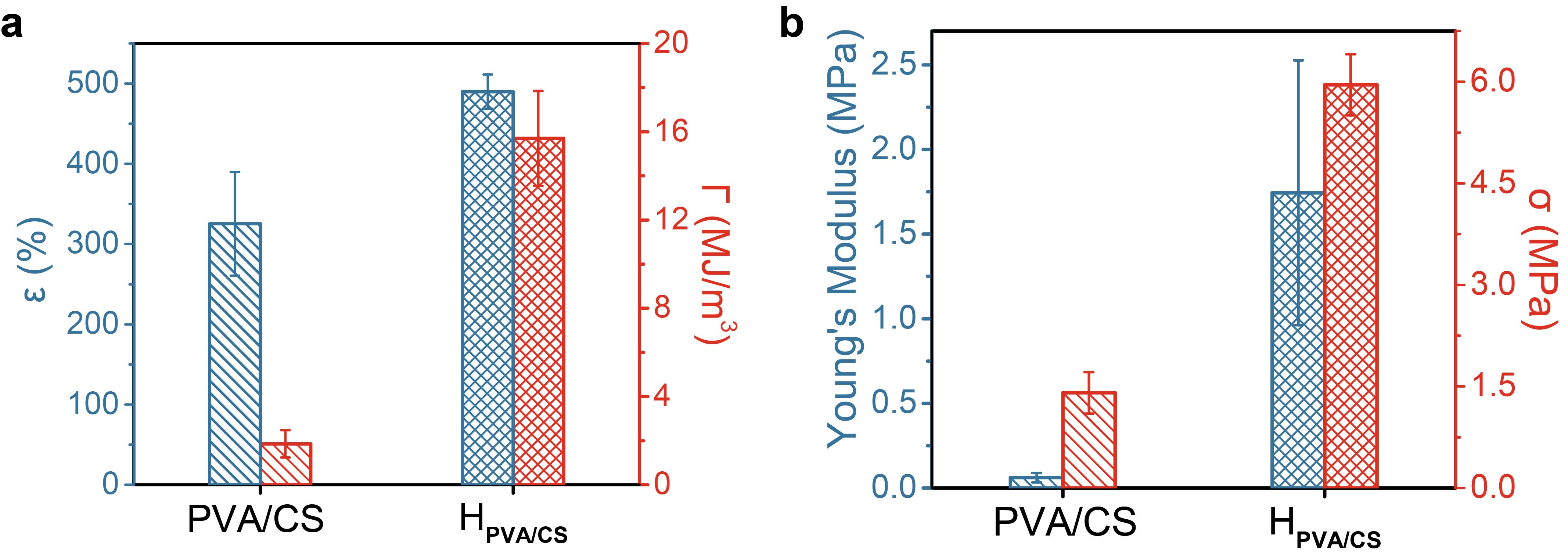


**Figure S7.** Intrinsic mechanical performance of PVA/CS and H_PVA/CS_ samples. (a) Tensile strain(ε) and toughness (Γ). (b) Young’ s modulus and Tensile strength(σ). Data in these figures are means ± SD, n = 3.


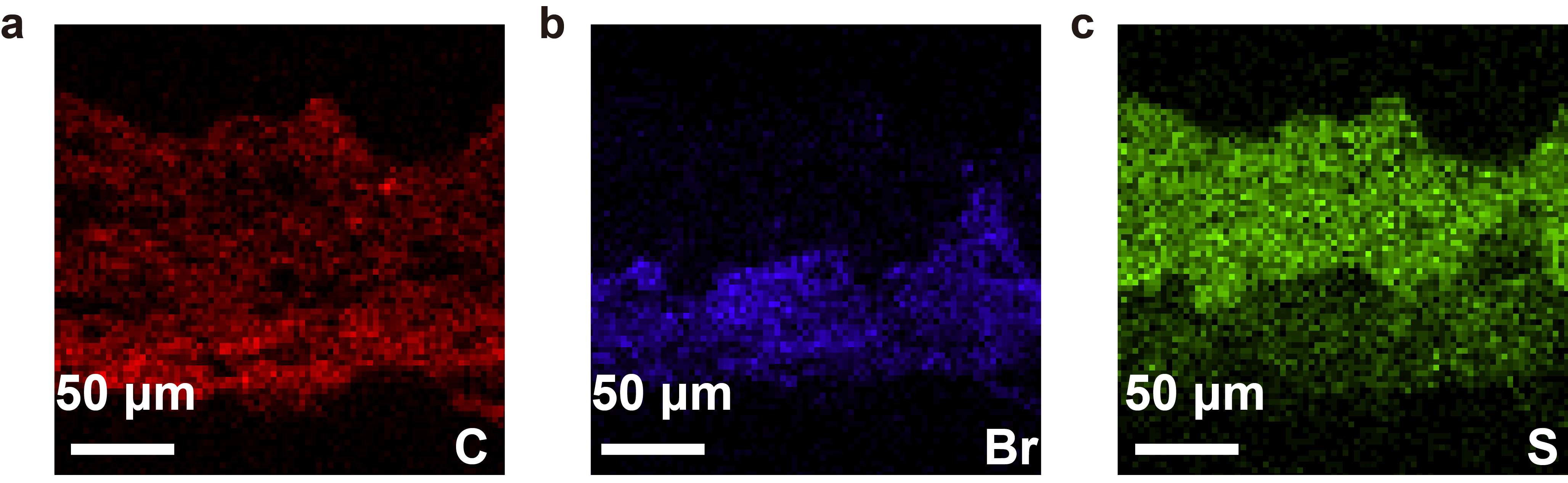


**Figure S8.** Cross-section EDS mapping (a, b and c) of C, Br and S elements for H_PVA/CS-PSPMA._ Scale bar: 50 μm.


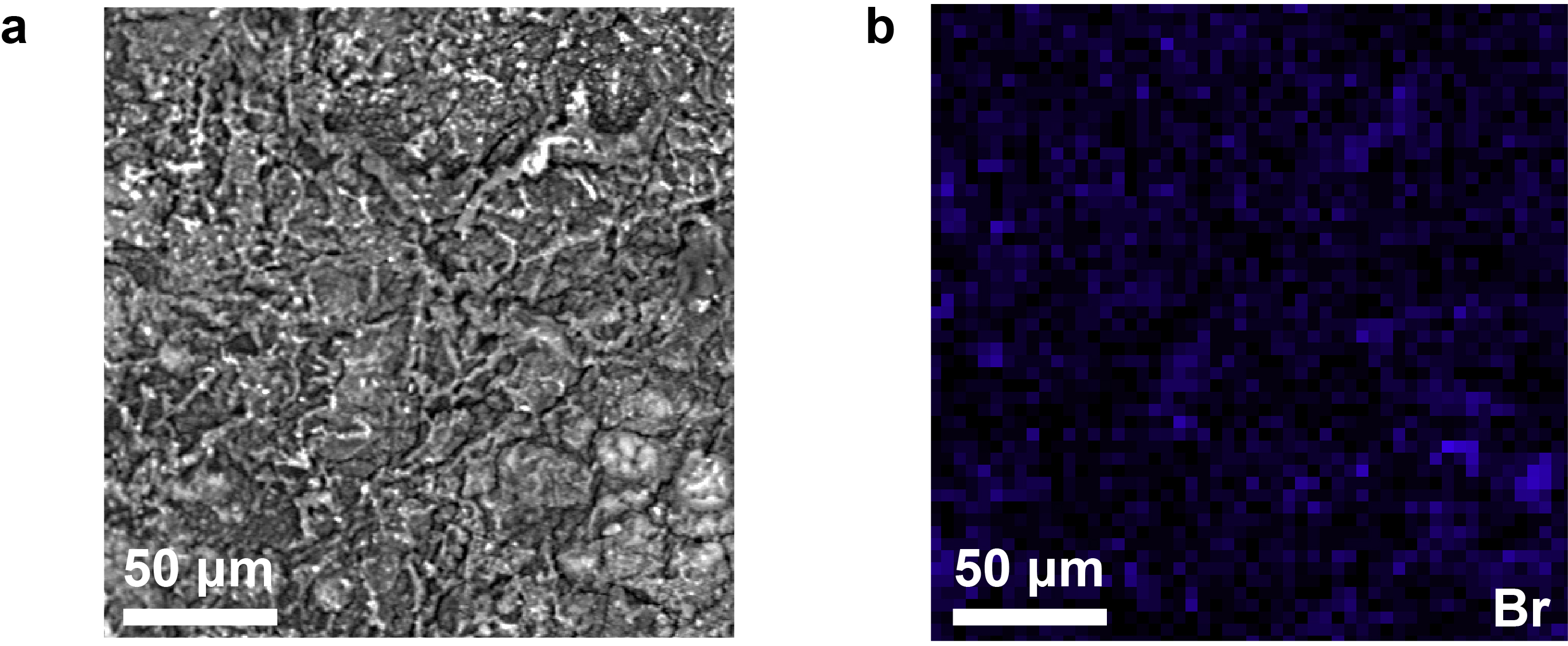


**Figure S9.** (a) Surface SEM image of the H_PVA/CS-Br_, and (b) corresponding EDS mapping spectrum of Br elements. Scale bar, 50μm.


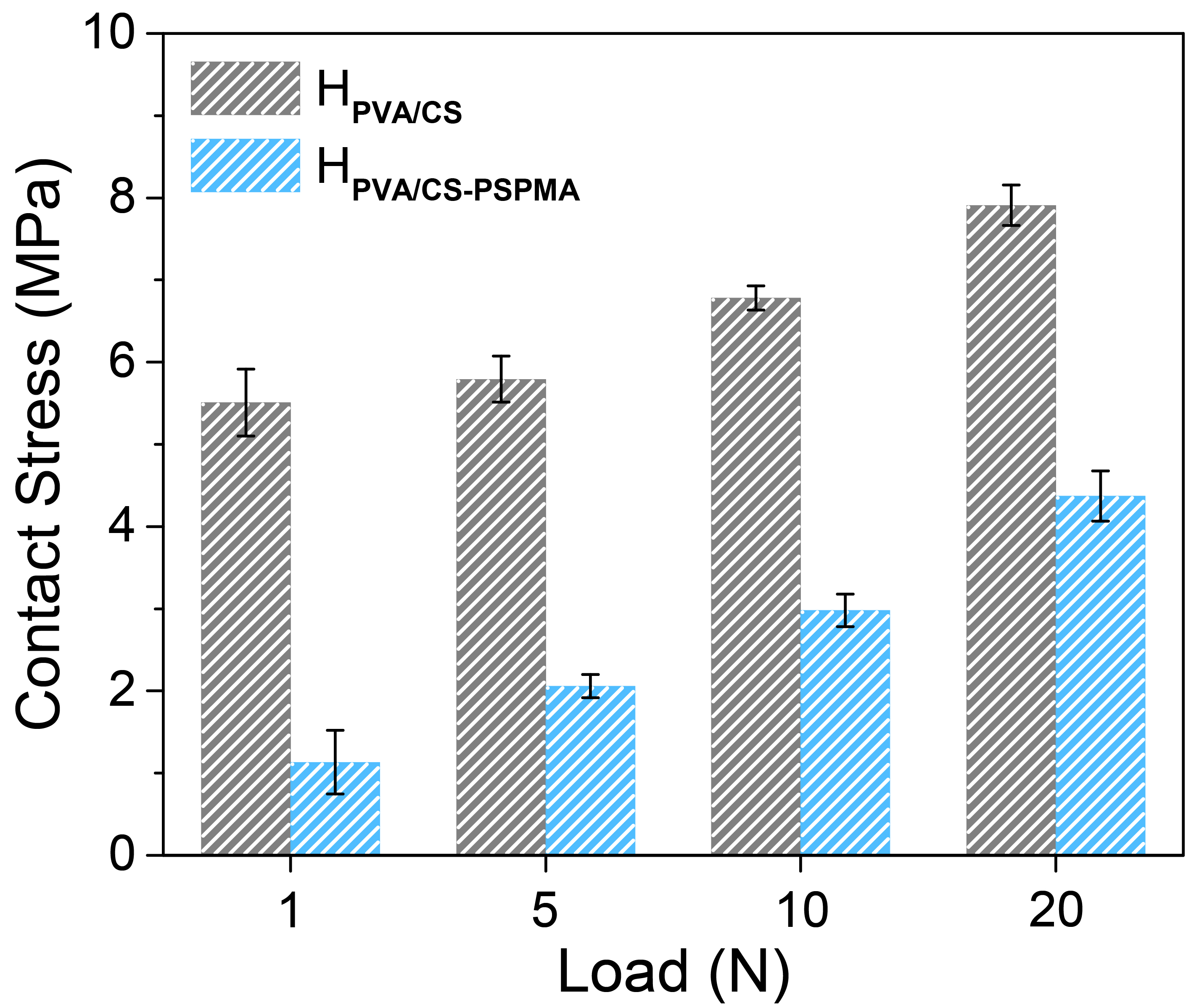


**Figure S10.**The calculated average contact stress values of H_PVA/CS_ and H_PVA/CS-PSPMA_ against steel ball under different normal loads. Data in this figure are means ± SD, n = 3.


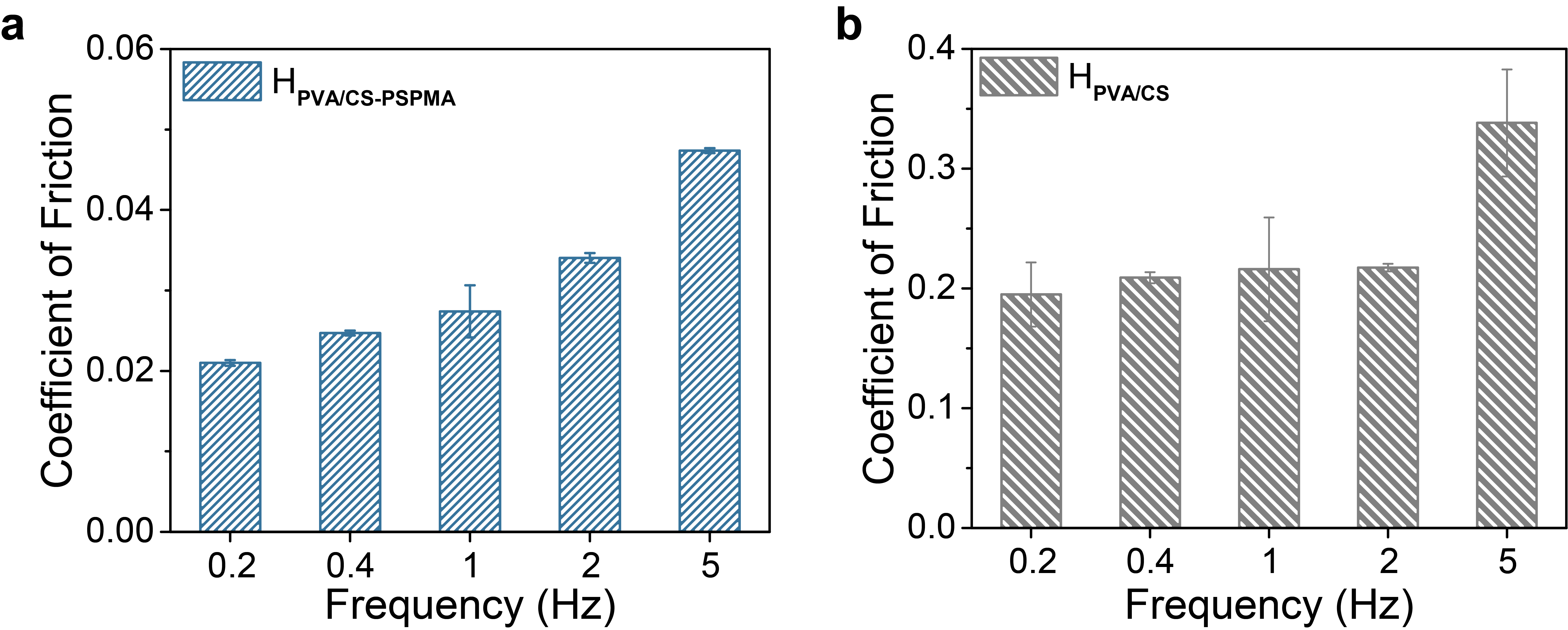


**Figure S11.** Average COFs of the H_PVA/CS-PSPMA_ (a) and H_PVA/CS_ (b) at different frequencies (5 N). Data in these figures are means ± SD, n = 3.


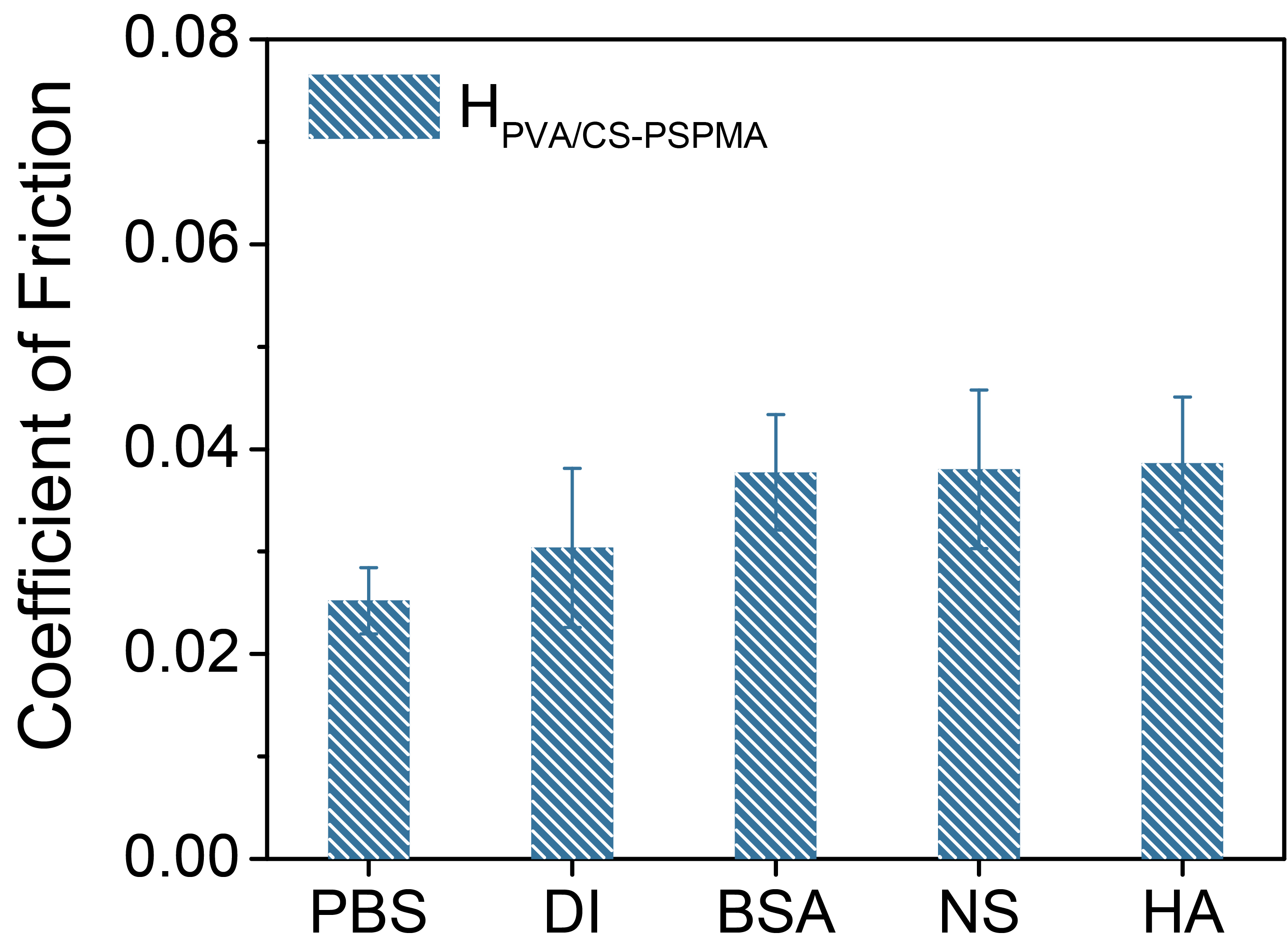


**Figure S12.** Average COFs of the H_PVA/CS-PSPMA_ samples with different lubricants (5 N). Data in this figure are means ± SD, n = 3.


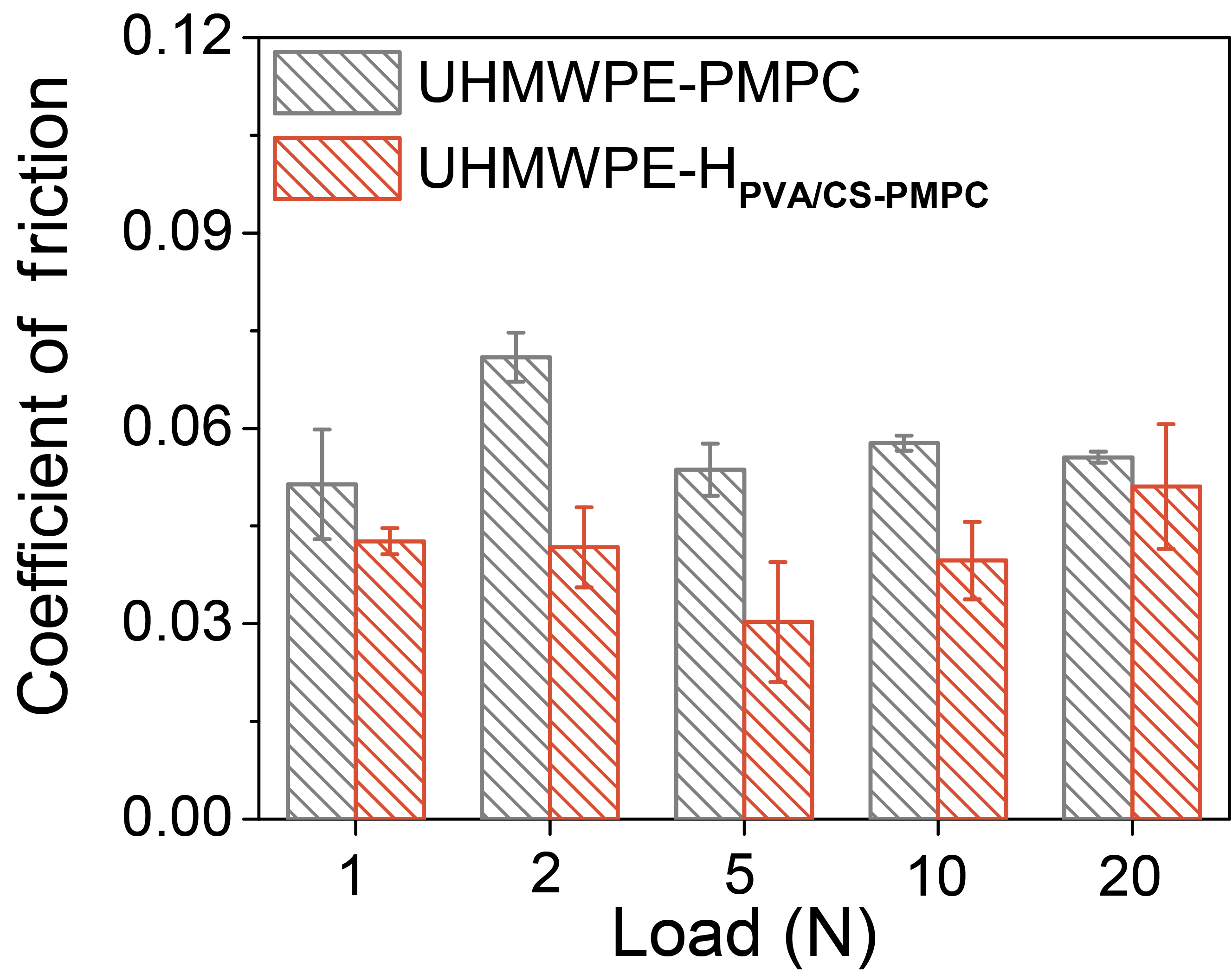


**Figure S13.** Average COFs values of UHMWPE-PMPC and UHMWPE-H_PVA/CS-PMPC_ under different normal loads (Frequency: 1 Hz, Lubricant: PBS) Data in this figure are means ± SD, n = 3.


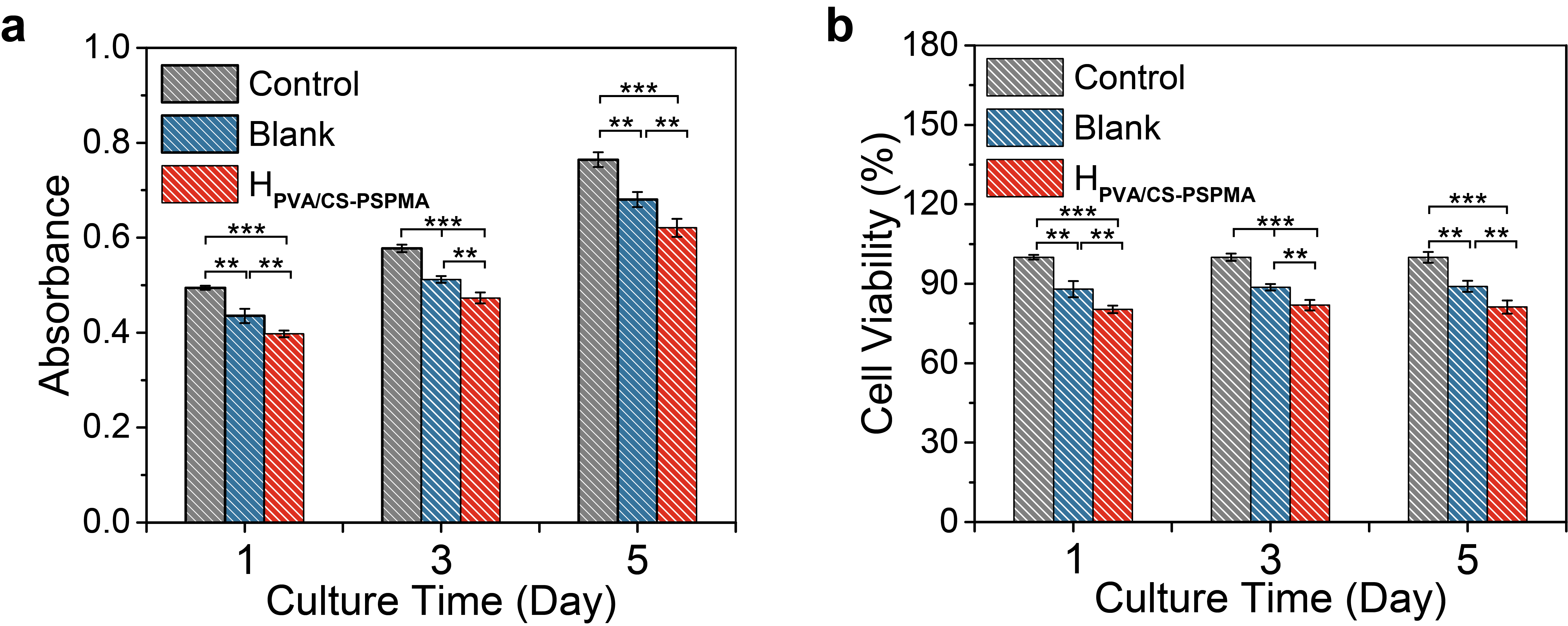


**Figure S14.** (a) Proliferation during cell culture. (b) Cell viability in five days by CCK-8 method. n=3, α=0.05, *p < 0.05, **p < 0.01, ***p < 0.001.


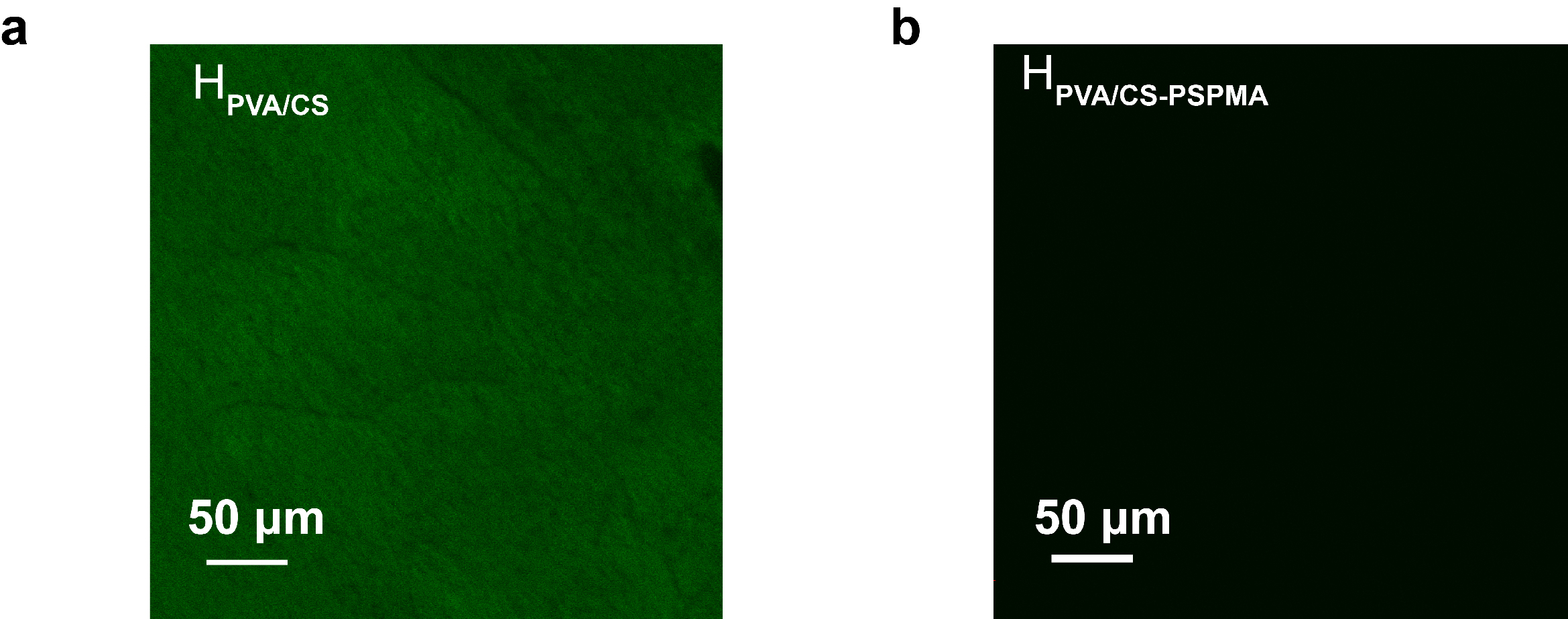


**Figure S15.** Representative CLSM fluorescent images of the controlled H_PVA/CS_ sample (a) with H_PVA/CS-PSPMA_ sample (b) in protein adsorption test. Scale bar, 50 μm.


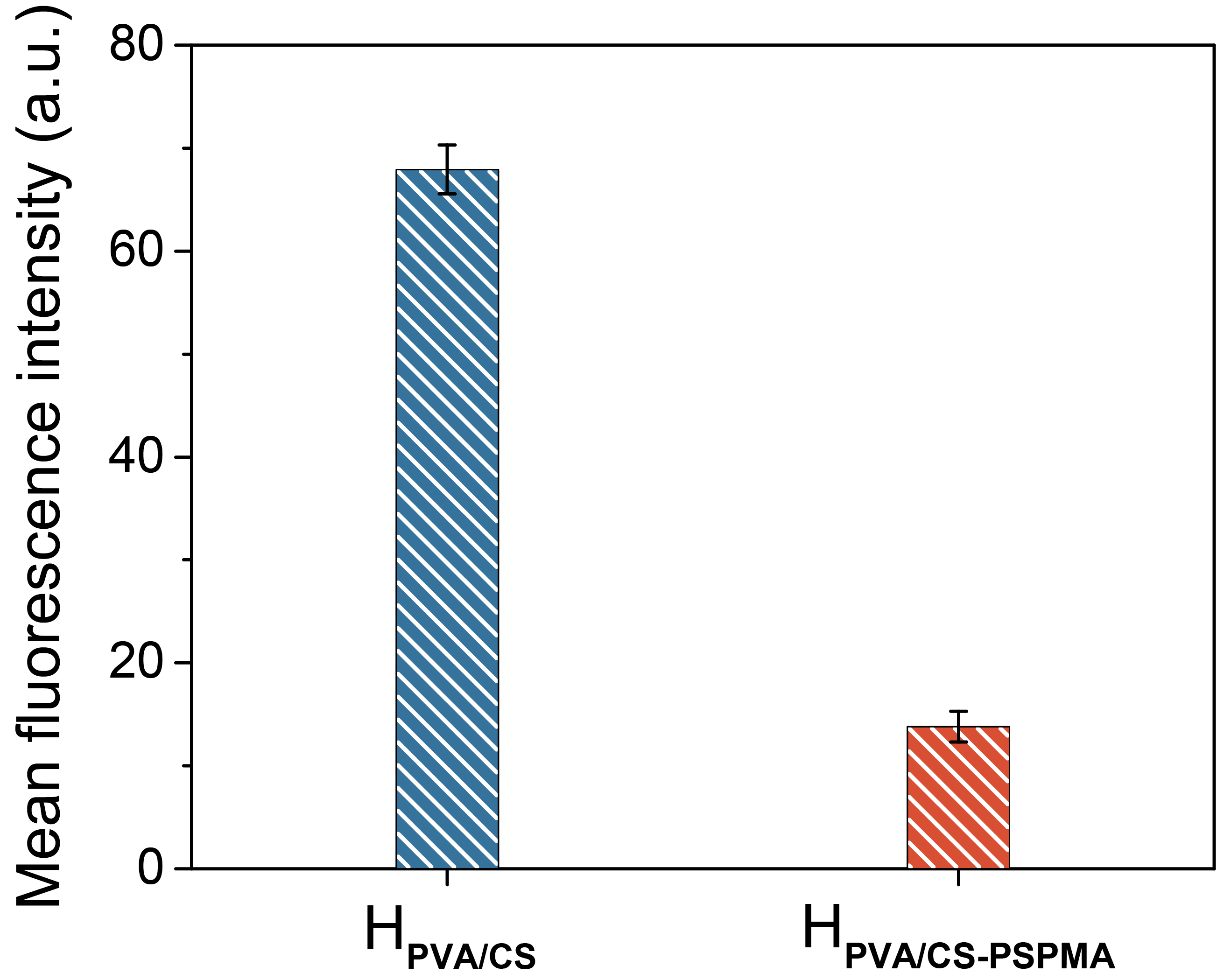


**Figure S16.** Mean fluorescence intensities of H_PVA/CS_ and H_PVA/CS-PSPMA_ surfaces. Data in this figure are means ± SD, n = 3.

**Supplemental Note 3.** **Reference**

[1] K. Ishihara, Y. Iwasaki, S. Ebihara, Y. Shindo, N. Nakabayashi, *Colloids Surf., B.* **2000**, 18, 325.

[2] J. Zhao, H. Tong, A. Kirillova, W. J. Koshut, A. Malek, N. C. Brigham, M. L. Becker, K. Gall, B. J. Wiley, *Adv. Funct. Mater.* **2022**, 32, 2205662.

[3] X. Liang, G. Chen, S. Lin, J. Zhang, L. Wang, P. Zhang, Z. Wang, Z. Wang, Y. Lan, Q. Ge, J. Liu, *Adv. Mater.* **2021**, 33, 2102011.

[4] M. Hua, S. Wu, Y. Ma, Y. Zhao, Z. Chen, I. Frenkel, J. Strzalka, H. Zhou, X. Zhu, X. He, *Nature* **2021**, 590, 594.

[5] V. L. Popov, *Contact mechanics and friction*, Springer, **2010**.

[6] S. Lin, J. Liu, X. Liu, X. Zhao, *Proc. Natl. Acad. Sci. U. S. A.* **2019**, 116, 10244.

[7] P. Jiang, P. Lin, C. Yang, H. Qin, X. Wang, F. Zhou, *Chem. Mater.* **2020**, 32, 9983.
